# Robotic-assisted lung transplantation: a systematic review of early outcomes in minimally invasive transplant surgery

**DOI:** 10.1007/s11701-026-03965-7

**Published:** 2026-09-19

**Authors:** Bilal Khan Mohammed, Aqsa Muskaan, Yahiya Pasha Quadri Syed, Abhishek Prasad, Mahin Sheikh, Teja Devarakonda, Akshay Kumar

**Affiliations:** 1 Division of Cardiac Surgery, Department of Surgery, Northwestern University Feinberg School of Medicine, 745 N Fairbanks Ct, Chicago, IL 60611 USA; 2 Canning Thoracic Institute, Northwestern Medicine, Chicago, IL USA; 3 Department of Medicine, Ayaan Institute of Medical Sciences, Hyderabad, India; 4 Department of Hospital Medicine, UW Health Swedish American Hospital, Rockford, IL USA; 5 Department of Anesthesiology and Perioperative Medicine, UT Health Medical Center, Texas Houston, USA; 6 Department of Cardiothoracic Surgery, University of California, San Francisco, CA USA; 7 Department of Cardiothoracic Surgery, NYU Langone Health, New York, NY USA

**Keywords:** Robotic surgery, Lung transplantation, Minimally invasive surgery, Primary graft dysfunction, Systematic review

## Abstract

Lung transplantation is conventionally performed through clamshell thoracosternotomy or sternotomy, approaches associated with pain, impaired wound healing, and chronic neuralgia. Robotic assistance has reached transplantation only recently. We systematically reviewed the clinical experience with robotic-assisted lung transplantation. Following PRISMA 2020 and PRISMA-S, we searched MEDLINE, Embase, Scopus, Web of Science, and Cochrane CENTRAL from inception to 15 August 2026, with trial registries, conference abstracts, and Chinese-language sources, without language restriction. Human robotic lung transplant reports with at least one clinical outcome were eligible. Because only one comparative cohort existed, synthesis was narrative; no meta-analysis was performed. Certainty was graded by outcome. PROSPERO: CRD420261479535. Out of 393 database records and 7 from other methods, six reports from five reporting centers (27 unique recipients) were included. No randomized trial or prior full-length systematic review was identified. In the only comparative cohort (robotic *n* = 21 vs. direct-vision minimally invasive *n* = 90), one-year survival was 95.0% versus 95.5% (log-rank *P* = 0.84) and grade-3 primary graft dysfunction at 72 h 4.8% versus 8.9% (odds ratio 0.51, 95% CI 0.06–4.34), despite longer ischemic times (486 vs. 408 min; *P* = 0.02). Every robotic versus direct-vision confidence interval included unity. Certainty was very low for all outcomes. Robotic lung transplantation is technically feasible across five reporting centers. Comparative evidence rests on one clinically selected, non-randomized cohort in which no difference was statistically detectable; this establishes neither equivalence nor absence of harm. Multicenter prospective evaluation with standardized outcomes is required.

**Registration:** The protocol was registered with PROSPERO (CRD420261479535).

**Supplementary Information:**

The online version contains supplementary material available at https://doi.org/10.1007/s11701-026-03965-7.

## Introduction

Lung transplantation remains the only definitive therapy for selected patients with end-stage pulmonary disease. Access is conventionally achieved through transverse thoracosternotomy (clamshell) or median sternotomy for bilateral grafts, or posterolateral thoracotomy for single-lung procedures [1]. These wide exposures are associated with post-operative pain, delayed wound healing, and chronic neuralgia [1, 2]. Primary graft dysfunction (PGD), the principal cause of early morbidity and mortality, is driven largely by ischemia–reperfusion injury and donor/recipient factors rather than by incision type [3, 4].

Robotic platforms have reshaped thoracic surgery: in lung cancer they now account for a growing share of anatomic resections, with short-term outcomes comparable to video-assisted thoracoscopic surgery [5, 6]. In transplantation, sternal-sparing approaches have been associated with reduced analgesia requirements and preserved early outcomes [2]. Robotic assistance extends these principles through tremor-filtered articulation, three-dimensional visualization, and wristed instrumentation that facilitate precise anastomoses through small, non–rib-spreading incisions [1, 7].

The translation of robotics to lung transplantation is nascent. The first robot-assisted lung transplant was performed in October 2021 and reported in 2024, followed by the first fully robotic procedures and a small number of single-center reports [7–9]. These milestones parallel the extension of robotics to other solid-organ transplants, including the first totally robotic heart transplant, and are increasingly framed within consensus efforts to standardize minimally invasive transplantation [10, 11]. Despite growing interest, the clinical evidence has not been systematically assembled. We therefore performed the first systematic review of robotic-assisted lung transplantation, synthesizing feasibility, perioperative, and early survival outcomes and appraising the strength and limitations of the current evidence.

## Methods

### Protocol and reporting

This review was conducted and reported in accordance with the PRISMA 2020 statement [12] and its literature-search extension, PRISMA-S [13]. The review protocol was registered with PROSPERO (registration number CRD420261479535). The additional analyses reported below were developed after the included studies were assembled, are not part of the registered protocol, and are therefore reported as exploratory and post hoc.

### Information sources and search strategy

We searched MEDLINE (PubMed), Embase, Scopus, Web of Science Core Collection, and Cochrane CENTRAL with the Cochrane Database of Systematic Reviews from inception to 15 August 2026, without language restriction, together with ClinicalTrials.gov, the WHO trials registry, conference abstracts (ISMICS, ISHLT, AATS, STS, EACTS), supplementary academic search engines, Google Scholar, and the Chinese-language literature. Institutional press releases informed narrative context only. Each strategy combined controlled vocabulary (MeSH/Emtree) and free-text terms for two concepts, robotic surgery and lung transplantation; full strategies and per-database yields are in Supplementary Appendix S1. Reference lists were screened and forward-citation searching performed on all included studies.

### Eligibility criteria

We included peer-reviewed reports of human robotic or robot-assisted lung transplantation describing perioperative technique with at least one clinical outcome (feasibility, perioperative morbidity, graft function, or survival). We excluded animal and cadaveric studies, purely descriptive technique reports without outcomes, robotic surgery performed in transplant recipients for other indications, and non-transplant procedures. No restriction was placed on laterality, indication, or robotic platform.

### Study selection and data extraction

Records were de-duplicated in the reference manager and screened by title and abstract, and full texts were assessed against the eligibility criteria. Screening, data extraction, and risk-of-bias appraisal were performed by a single reviewer; independent duplicate screening was not undertaken, and this is acknowledged as a limitation. Extracted values were verified against the source publications in a second pass. The full electronic search strategies, per-database yields, de-duplication summary, and the citations and reasons for both full-text exclusions are provided in the Supplementary Material. From each study we extracted center, period, design, recipient number, laterality, indication, platform, incision and port configuration, insufflation, anastomotic sequence and times, suture material, bedside assistance, ischemic times, conversion events, mechanical ventilation, length of stay, PGD, and survival. Where reported, we also extracted the basis on which recipients were selected for the robotic approach (prior thoracic surgery or anticipated adhesions, recipient acuity, pre-transplant bridging support, and anatomical or physiological considerations) and the intraoperative extracorporeal-support strategy.

### Risk of bias assessment

Five included reports were non-comparative: two single-patient case reports (Jiao 2023; Park 2026) and three case series (Emerson 2024, eight patients; Chang 2025, two patients; Ascanio 2024, two patients). The Joanna Briggs Institute Checklist for Case Reports was applied to the former and the Checklist for Case Series to the latter. These critical-appraisal tools assess methodological quality and risk of bias; item-level judgments are reported without a numeric summary score. The single comparative cohort was appraised using the ROBINS-I tool for non-randomized studies of interventions. Certainty of evidence was assessed outcome by outcome with the GRADE approach adapted to narrative synthesis, which does not require a pooled estimate [14].

### Synthesis and statistical methods

Because only one study included a concurrent non-robotic comparator, quantitative meta-analysis was neither valid nor performed; results were synthesized narratively and in structured tables. For the single comparative cohort, odds ratios with 95% confidence intervals were computed for dichotomous outcomes using the Haldane–Anscombe correction where a zero cell was present; exact (Clopper–Pearson) 95% confidence intervals were computed for proportions in the largest single-center robotic cohort. Confidence intervals for odds ratios were computed by the Woolf (log) method, with the Haldane–Anscombe continuity correction applied only when a zero cell was present; exact binomial intervals were used for single proportions. P values presented in the comparative outcomes table are those reported by the source publication, whereas the P values displayed in the forest plot are two-sided Fisher exact tests recomputed from the displayed counts so that the test corresponds to the tabulated 2 × 2 data; where the two differ, both are shown. No adjustment was made for multiplicity, so P values are descriptive rather than confirmatory. The ventilation outcome is presented consistently as ventilator support for less than 48 h throughout. No test of non-inferiority or equivalence was undertaken, and absence of a statistically significant difference is not interpreted as equivalence.

### Individual patient data and additional analyses

Where reports published patient-level detail we extracted individual patient data (age, sex, indication, laterality, ischemic times, serial PGD grade) to permit analyses not possible from summary statistics. The eight patient-level cases in the first-in-man report are a subset of the 21-patient cohort from the same center and were not counted separately; 27 unique recipients have been reported, of whom 14 have patient-level data. Three additional analyses were developed after the studies were assembled and are therefore exploratory and post hoc: ordinary least-squares regression of warm ischemic time on consecutive case number; unadjusted odds ratios for grade-3 PGD across all three access strategies, the comparative cohort having also reported a contemporaneous standard-access group (*n* = 98); and Spearman correlation of ischemic duration with graft function. Computations used Python 3 (SciPy).

## Results

### Study selection

**Fig. 1:**
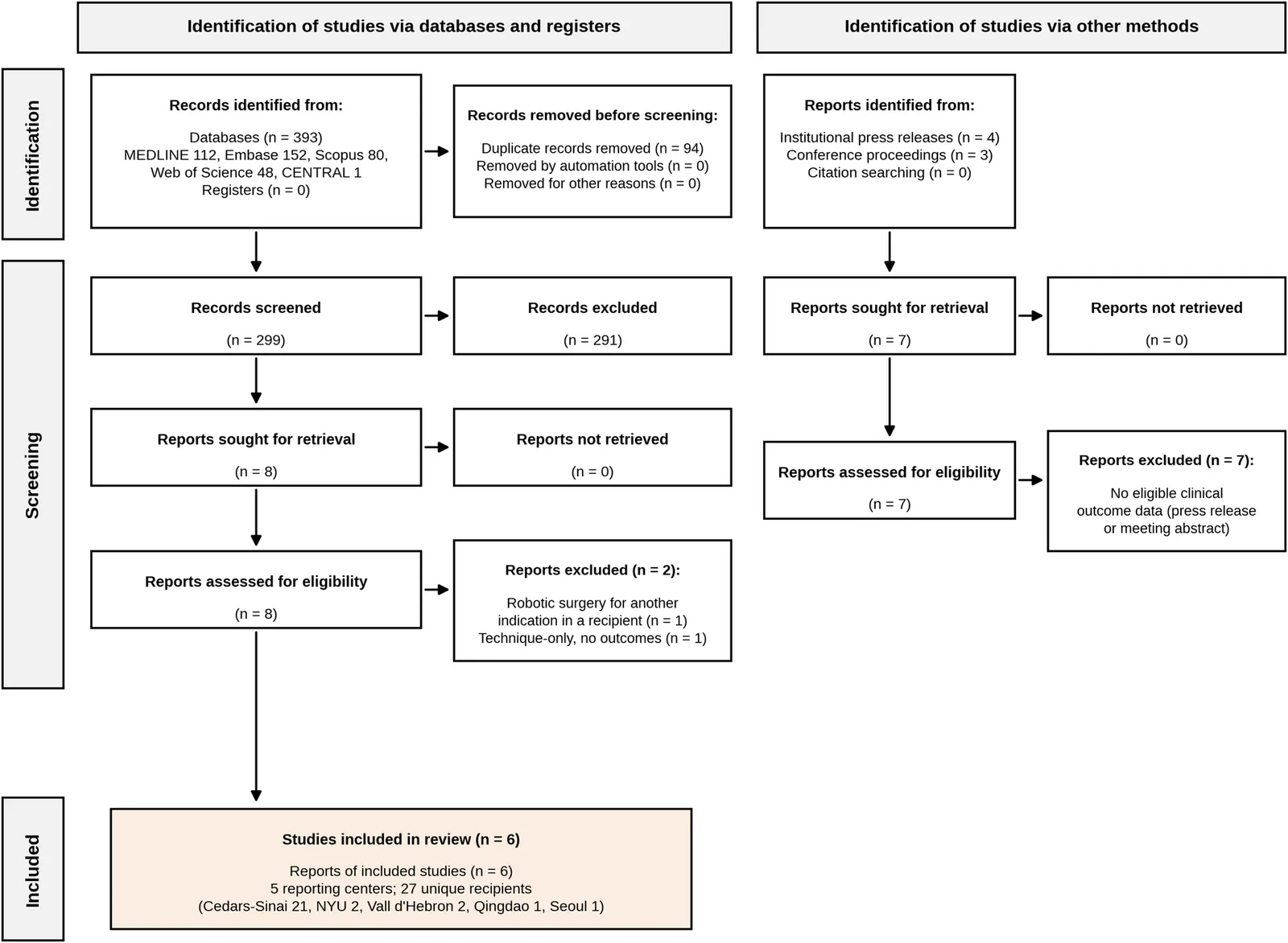
PRISMA 2020 flow diagram, drawn to the official two-stream template. Database and register searching yielded 393 records; 94 duplicates were removed, 299 records were screened, 291 excluded, and 8 reports assessed for eligibility, of which 2 were excluded. Other methods (institutional press releases and conference proceedings) yielded 7 records, all excluded for absence of eligible clinical outcome data. Six reports from five reporting centers and 27 unique recipients were included. Full search strategies, per-database yields, and full-text exclusion records are provided in the Supplementary Material

Database searching yielded 393 records and other methods a further 7. After removal of 94 duplicates, 299 database records were screened and 8 reports assessed in full text, of which 6 met the inclusion criteria; none of the 7 records identified by other methods yielded an eligible report (Fig. 1). No randomized or controlled trial of robotic lung transplantation was identified in CENTRAL, ClinicalTrials.gov, or the WHO registry—the single CENTRAL record was a conference-proceedings entry without outcome data—and no prior full-length peer-reviewed systematic review, Cochrane review, eligible preprint, or additional non-English clinical report was found. The two excluded full texts were a report of robotic surgery for another indication in a recipient and a standardized-technique report without separately identifiable patient outcomes. The same group’s sheep-model validation study and companion editorial were also assessed and excluded, as animal research and as an opinion piece without patient-level outcomes respectively.

### Included studies and centers

The six reports originated from five reporting programs across North America, Asia, and Europe (Table 1): Cedars-Sinai Medical Center (first robot-assisted transplant, performed in October 2021 and reported in 2024, and a subsequent comparative cohort), the Affiliated Hospital of Qingdao University, New York University Langone (first fully robotic bilateral transplant), Seoul National University, and Hospital Universitario Vall d’Hebron, Barcelona, which reported two recipients implanted through a subxiphoid incision. Robotic platforms were predominantly the da Vinci system, and anastomoses were performed in a bronchus-first sequence in most reports [1, 8].

**Table 1:** Included studies

Study (year)	Center, Country	Design	*n*	Laterality	Indication	Platform	Key outcome
Emerson 2024 [8]	Cedars-Sinai, USA	Case series (first-in-man + 7)	8	Single and bilateral	COPD, IPF	da Vinci	First robot-assisted LTx; no grade-3 PGD among reported 24/48-h grades
Jiao 2023 [1]	Qingdao, China	Case report	1	Right single	COPD	da Vinci Si	Extubated 36 h; discharged POD-19; FEV1 0.27→1.01 L
Emerson 2025 [16]	Cedars-Sinai, USA	Comparative cohort (not matched)	21 (vs. 90)	62% bilateral	Fibrosis predominant	da Vinci	1-yr survival 95.0% vs. 95.5% (*P* = 0.84)
Chang 2025 [9]	NYU Langone, USA	Case series	2	Bilateral	COPD/emphysema	da Vinci Xi	First fully robotic bilateral LTx; PGD 0 in both
Park 2026 [15]	Seoul National, Korea	Case report	1	Right robotic + left VATS	Idiopathic pulmonary fibrosis	da Vinci Xi	First assistant-independent robotic implantation (right lung)
Ascanio 2024 [20]	Vall d’Hebron, Spain	Case series	2	Single lung (left, case 2)	Usual interstitial pneumonia; pulmonary fibrosis	Not specified	Fully robotic via subxiphoid access; minimal pain, no opioids, no extracorporeal support

The six reports come from five reporting centers and 27 unique recipients (Cedars-Sinai 21, NYU 2, Vall d’Hebron 2, Qingdao 1, Seoul 1); the eight patient-level cases in Emerson 2024 are a subset of the 21-patient Cedars-Sinai cohort and are not counted separately. The Vall d’Hebron report is designated an Editorial by its journal but reports two human recipients with donor and recipient characteristics, operative technique, and post-operative clinical outcomes, and was therefore included on content rather than on article-type label. That group’s sheep-model study, standardized-technique paper, and companion editorial were assessed and excluded (Supplementary Material S3). COPD, chronic obstructive pulmonary disease; LTx, lung transplantation; VATS, video-assisted thoracoscopic surgery

### Risk of bias and certainty

The three-case series met most Joanna Briggs Institute items, incomplete outcome reporting the recurrent shortfall (Table 2); both case reports met all eight items (Table 3). The comparative cohort carried serious risk of bias by ROBINS-I, driven by confounding by indication and clinical selection. Certainty was very low for every outcome (Table 4).

**Table 2:** Critical appraisal of the three included case series (Joanna Briggs Institute Checklist for Case Series, 10 items)

JBI case-series item	Emerson 2024 [8] (*n* = 8)	Chang 2025 [9] (*n* = 2)	Ascanio 2024 [20] (*n* = 2)
1. Clear criteria for inclusion in the case series	Unclear	Yes	Unclear
2. Condition measured in a standard, reliable way for all participants	Yes	Yes	Yes
3. Valid methods used for identification of the condition	Yes	Yes	Yes
4. Consecutive inclusion of participants	Yes	Yes	Yes
5. Complete inclusion of participants	Yes	Yes	Yes
6. Clear reporting of participant demographics	Yes	Yes	Yes
7. Clear reporting of clinical information	Yes	Yes	Yes
8. Outcomes or follow-up results clearly reported	No (72-h PGD not reported for all)	Yes	No (no graft-function or survival data)
9. Clear reporting of presenting site/clinic demographic information	Yes	Yes	Yes
10. Statistical analysis appropriate	Not applicable (descriptive)	Not applicable (descriptive)	Not applicable (descriptive)

Appraisal performed with the current Joanna Briggs Institute Checklist for Case Series [25]. Appraisal was undertaken by a single reviewer; independent duplicate appraisal was not performed. These critical-appraisal tools assess methodological quality and risk of bias; item-level judgments are reported without a numeric summary score

**Table 3:** Critical appraisal of the two included case reports (Joanna Briggs Institute Checklist for Case Reports, 8 items)

JBI case-report item	Jiao 2023 [1]	Park 2026 [15]
1. Patient demographic characteristics clearly described	Yes	Yes
2. Patient history clearly described and presented as a timeline	Yes	Yes
3. Current clinical condition on presentation clearly described	Yes	Yes
4. Diagnostic tests or assessment methods and results clearly described	Yes	Yes
5. Intervention or treatment procedure clearly described	Yes	Yes
6. Post-intervention clinical condition clearly described	Yes	Yes
7. Adverse events or unanticipated events identified and described	Yes	Yes
8. Takeaway lessons provided	Yes	Yes

Appraisal performed with the current Joanna Briggs Institute Checklist for Case Reports [25]. The single comparative cohort (Emerson 2025 [16]) was appraised with ROBINS-I [26]: overall serious risk of bias, driven by confounding by indication and selection of participants (non-randomized, single-center, clinically selected, small n); outcome ascertainment (primary graft dysfunction grade, survival) was objective. A fully reported case series still provides weak causal evidence because its design cannot control confounding

**Table 4:** GRADE certainty of evidence, by outcome (narrative synthesis; no pooled estimate)

Outcome	Contributing evidence	Risk of bias	Inconsistency	Indirectness	Imprecision	Certainty
One-year survival	1 non-randomized comparative cohort (21 vs. 90)	Serious	Not assessable (single study)	Not serious	Very serious (few events; CI includes unity)	Very low
Grade-3 PGD at 72 h	1 non-randomized comparative cohort	Serious	Not assessable	Not serious	Very serious (1 vs. 8 events)	Very low
Ventilator support < 48 h	1 non-randomized comparative cohort	Serious	Not assessable	Not serious	Very serious	Very low
30-day death	1 non-randomized comparative cohort	Serious	Not assessable	Not serious	Very serious (1 vs. 0 events)	Very low
Technical feasibility / completion	3 case series, 2 case reports, 1 cohort	Very serious (non-comparative)	Not assessable	Not serious	Serious	Very low

Certainty assessed with the GRADE approach applied to narrative synthesis [14]. All bodies of evidence begin at low certainty because every included study is non-randomized, and all were further downgraded for serious risk of bias and very serious imprecision, yielding very low certainty for every outcome. Certainty was not rated up because no large effect, dose-response gradient, or opposing plausible confounding was identified. Inconsistency could not be assessed for comparative outcomes because only one comparative cohort exists. PGD, primary graft dysfunction

### Feasibility and technique

Across reports, the bronchial, arterial, and atrial anastomoses were completed with robotic instrumentation and the allograft delivered through a utility incision without rib spreading [1]. In the single-case Chinese report, a right single-lung transplant in a 59-year-old man with chronic obstructive pulmonary disease and dense pleural adhesions required bronchial, arterial, and atrial anastomotic times of 12, 28, and 32 min; cold ischemic time was 300 min, extubation occurred at 36 h, discharge on postoperative day 19, and FEV1 improved from 0.27 to 1.01 L [1].

The Seoul group reported the first assistant-independent robotic implantation: in a 68-year-old man with idiopathic pulmonary fibrosis, the right lung was implanted robotically without bedside suture handling (cold and warm ischemic times 349 and 131 min) and the left by video-assisted thoracoscopy. Grade-3 primary graft dysfunction at 24 and 48 h resolved to grade 0 by 72 h; 3-month FEV1 was 104% predicted [18].

### Comparative outcomes

**Fig. 2:**
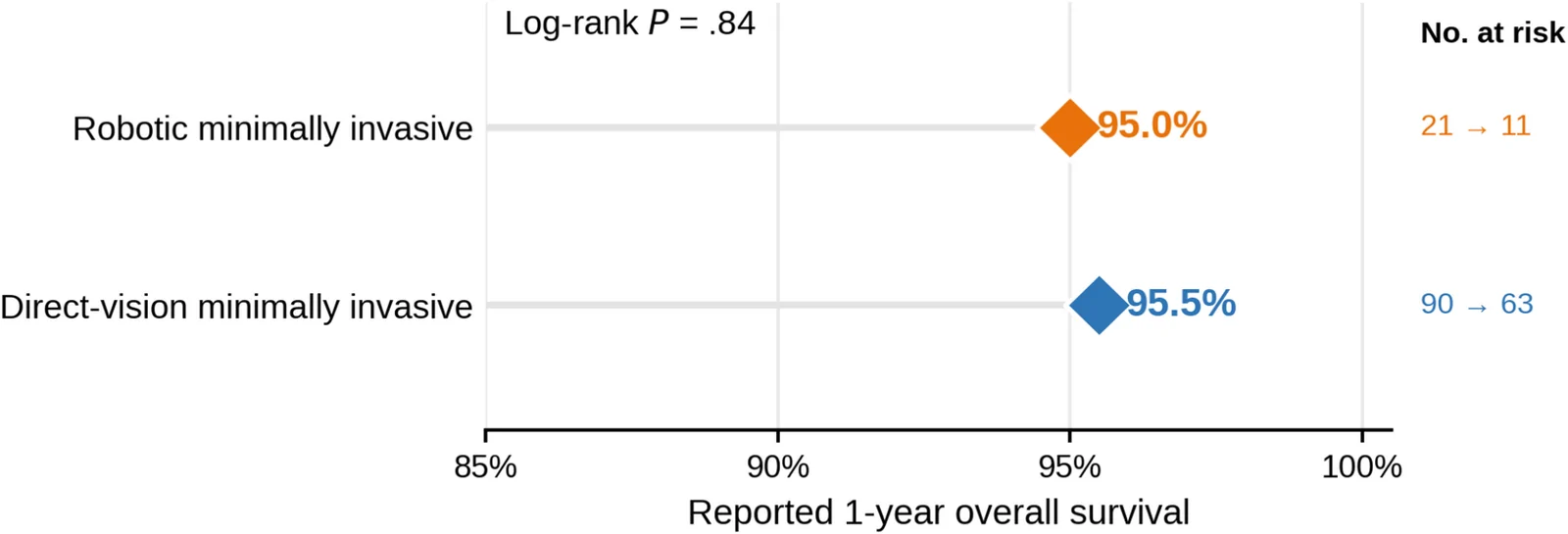
Reported one-year overall survival after robotic (95.0%) and direct-vision minimally invasive (95.5%) lung transplantation, with numbers at risk at baseline and 12 months (log-rank *P* = 0.84, as reported by the source). Individual event and censoring times were not published, so a Kaplan-Meier curve and its confidence bands cannot be reconstructed and are not presented

**Fig. 3:**
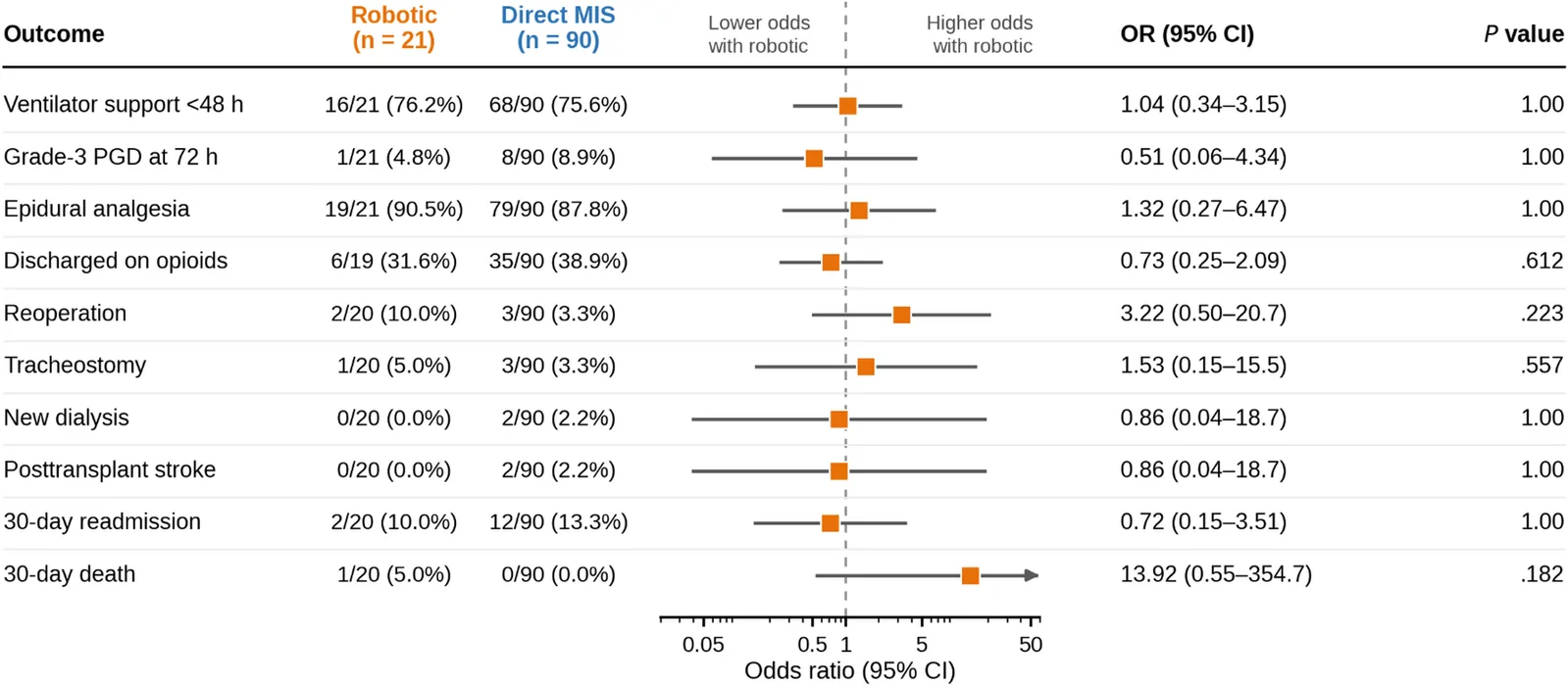
Forest plot of computed effect estimates for all reported in-hospital and 30-day outcomes, robotic versus direct-vision minimally invasive lung transplantation. Squares are odds ratios and horizontal lines 95% confidence intervals; the arrow denotes an interval extending beyond the plotted axis. Confidence intervals were computed by the Woolf method and the Haldane-Anscombe correction applied only where a zero cell was present; *P* values are two-sided Fisher exact tests recomputed from the displayed counts so that test and counts correspond, without adjustment for multiplicity. All ten confidence intervals include unity and no outcome is statistically distinguishable. For 30-day death (1/20 vs. 0/90) the source reports *P* = 0.03, whereas the exact test on those counts gives *P* = 0.18. No pooled estimate is presented because only one comparative cohort exists. MIS, minimally invasive surgery; OR, odds ratio; PGD, primary graft dysfunction

**Fig. 4:**
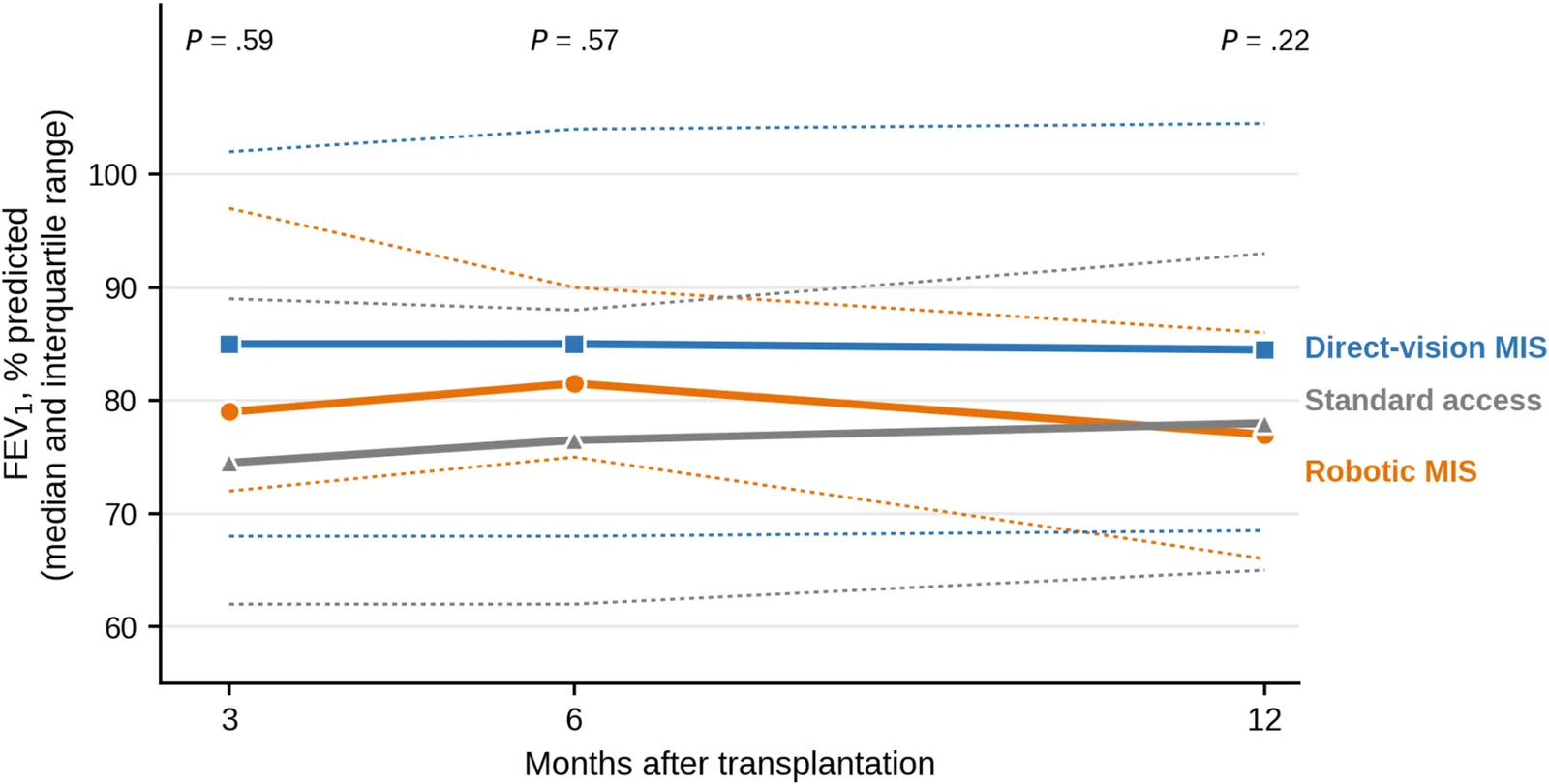
Pulmonary function recovery. Median FEV1 (% predicted) with interquartile range (dotted lines) at 3, 6, and 12 months after transplantation. *P* values are from the source repeated-measures linear mixed-effects model, which compared the robotic and direct-vision minimally invasive groups only; no difference reached statistical significance. The standard-access series is shown for descriptive context and was not included in that model, so no formal three-way inference is supported. MIS, minimally invasive surgery

Only the Cedars-Sinai cohort provided a concurrent non-robotic comparator, contrasting robotic (*n* = 21) with direct-vision minimally invasive lung transplantation (*n* = 90) on an intention-to-treat basis (Table 5; Figs. 2 and 3) [16]. One-year survival was 95.0% versus 95.5% (log-rank *P* = 0.84; Fig. 2). Grade-3 PGD at 72 h occurred in 4.8% (1/21) versus 8.9% (8/90) (OR 0.51, 95% CI 0.06–4.34; *P* = 0.53), and ventilator support for less than 48 h did not differ significantly (76.2% vs. 75.6%; OR 1.04, 95% CI 0.34–3.15). Median length of stay did not differ significantly (14.1 vs. 14.3 days; *P* = 0.95). Allograft ischemic time was longer in the robotic arm (median 486 vs. 408 min; *P* = 0.02; the source abstract states 406 min, whereas its Results and Table 5 state 408), as was waitlist time (50 vs. 22.5 days; *P* = 0.02); one early death occurred in the robotic group (1/20; 5.0%). The source reports *P* = 0.03 for this comparison, but a two-sided Fisher exact test on the displayed counts (1/20 vs. 0/90) gives *P* = 0.18, and the corresponding odds ratio is 13.92 (95% CI 0.55–354.75); the interval includes unity. Lung-function indices at 3, 6, and 12 months did not differ significantly between groups (Fig. 4). Across all ten reported in-hospital and 30-day outcomes, every computed confidence interval included unity and no comparison was statistically distinguishable when tested by two-sided Fisher exact tests matched to the displayed counts (Fig. 3).

**Table 5:** Comparative early outcomes — robotic versus direct-vision minimally invasive transplantation (Cedars-Sinai; intention-to-treat)

Outcome	Robotic (*n* = 21)	Direct-vision MIS (*n* = 90)	Effect (95% CI)	*P*
One-year survival	95.0%	95.5%	—	0.84 (log-rank)
Grade-3 PGD at 72 h	4.8% (1/21)	8.9% (8/90)	OR 0.51 (0.06–4.34)	0.53†; 1.00‡
Ventilator support < 48 h*	76.2% (16/21)	75.6% (68/90)	OR 1.04 (0.34–3.15)	0.79†; 1.00‡
Median length of stay, days	14.1	14.3	—	0.95
Allograft ischemic time, min	486	408	—	0.02
Waitlist time, days	50	22.5	—	0.02
Early death (30-day)	5.0% (1/20)	0% (0/90)	OR 13.92 (0.55–354.75)	0.03†; 0.18‡

*The ventilation outcome is presented throughout as ventilator support for less than 48 hours. Odds ratios were computed by the Woolf method with the Haldane–Anscombe correction only where a zero cell was present. †P value as reported by the source

‡Two-sided Fisher exact test recomputed from the displayed counts, without adjustment for multiplicity. The source abstract reports an ischemic time of 406 minutes whereas its Results and Table 2 report 408; the tabled value is used here. All comparative confidence intervals include unity. MIS, minimally invasive surgery; PGD, primary graft dysfunction

### Feasibility cohort

In the largest single-center robotic experience (*n* = 21; Table 6), 61.9% of procedures were bilateral and pulmonary fibrosis was the leading indication (57.1%). Intraoperative veno-arterial extracorporeal membrane oxygenation (VA-ECMO) was used in 42.9%; unplanned VA-ECMO conversion and conversion from a robotic to a nonrobotic approach each occurred in 14.3% (95% CI 3.0–36.3), and no conversion required incision enlargement. Freedom from grade-3 PGD at 72 h was 95.2% (95% CI 76.2–99.9), median warm ischemic time 68.5 min, median time to extubation 1.5 days, and 71.4% were discharged home. Survival over the study window was 90.5% (95% CI 69.6–98.8); both deaths were attributable to gastrointestinal hemorrhage and disseminated mucormycosis rather than graft failure.

**Table 6:** Descriptive feasibility outcomes — largest single-center robotic cohort (*n* = 21), exact (Clopper–Pearson) 95% confidence intervals

Variable	Value	95% CI
Bilateral transplant	61.9% (13/21)	38.4–81.9
Pulmonary fibrosis indication	57.1% (12/21)	34.0–78.2
Any intraoperative VA-ECMO	42.9% (9/21)	21.8–66.0
Unplanned VA-ECMO conversion	14.3% (3/21)	3.0–36.3
Robotic-to-nonrobotic conversion	14.3% (3/21)	3.0–36.3
Incision enlargement	0% (0/21)	0.0–16.1
Freedom from grade-3 PGD at 72 h	95.2% (20/21)	76.2–99.9
Discharged home	71.4% (15/21)	47.8–88.7
Survival over study window	90.5% (19/21)	69.6–98.8
Median warm ischemic (implant) time	68.5 min	—
Median time to extubation	1.5 days	—
Median length of stay	14.5 days	—

*VA-ECMO*, veno-arterial extracorporeal membrane oxygenation; *PGD*, primary graft dysfunction

### Pooled individual patient data

Fourteen of the 27 reported recipients had patient-level data (Table 7): median age 66.5 years (range 58–73), 10 men, with chronic obstructive pulmonary disease in 8 and interstitial lung disease or pulmonary fibrosis in 6. Anastomotic times were short relative to total implantation time (bronchial 12, arterial 28, atrial 32 min in the Chinese report [1]), whereas implantation took 131 min in the Seoul case [18] and exceeded 60 min in the Cedars-Sinai cohort [16].

**Table 7:** Pooled individual patient data — all published robotic lung transplant recipients with patient-level reporting

No.	Center	Source	Age	Sex	Indication	Procedure	Warm ischemia *R*/L, min	24 h	48 h	72 h
1	Cedars-Sinai	Emerson 2024 [8]	69	M	COPD	Right single	88 / —	1	0	0
2	Cedars-Sinai	Emerson 2024 [8]	63	M	IPF	Right single	111 / —	0	0	NR
3	Cedars-Sinai	Emerson 2024 [8]	73	F	COPD	Right single	85 / —	1	1	NR
4	Cedars-Sinai	Emerson 2024 [8]	67	M	COPD	Bilateral	68 / 86	1	0	NR
5	Cedars-Sinai	Emerson 2024 [8]	71	F	COPD	Bilateral*	81 / NA	2	2	NR
6	Cedars-Sinai	Emerson 2024 [8]	66	M	COPD	Right single	58 / —	0	1	NR
7	Cedars-Sinai	Emerson 2024 [8]	64	F	IPF	Bilateral	67 / 78	1	1	NR
8	Cedars-Sinai	Emerson 2024 [8]	66	M	IPF	Right single	66 / —	0	0	NR
9	Qingdao	Jiao 2023 [1]	59	M	COPD	Right single	NR	NR	NR	NR
10	NYU Langone	Chang 2025 [9]	58	F	COPD	Bilateral	70 / 74	0	NR	0
11	NYU Langone	Chang 2025 [9]	73	M	COPD	Bilateral	72 / 88	0	NR	0
12	Seoul National	Park 2026 [15]	68	M	IPF	Right robotic + left VATS	131 / 41	3	3	0
13	Vall d’Hebron	Ascanio 2024 [20]	65	M	UIP	Single lung	NR	NR	NR	NR
14	Vall d’Hebron	Ascanio 2024 [20]	71	M	Pulmonary fibrosis	Left single	NR	NR	NR	NR

*Only the right lung was implanted with robotic assistance. Cases 1–8 are a subset of the 21-patient cohort reported subsequently by the same center and are not counted separately; across the included literature 27 unique recipients have been reported, of whom 14 have patient-level data. Median age 66.5 years (range 58–73). COPD, chronic obstructive pulmonary disease; IPF, idiopathic pulmonary fibrosis; NR, not reported; PGD, primary graft dysfunction; R/L, right/left; VATS, video-assisted thoracoscopic surgery

### Learning curve (exploratory post hoc)

**Fig. 5:**
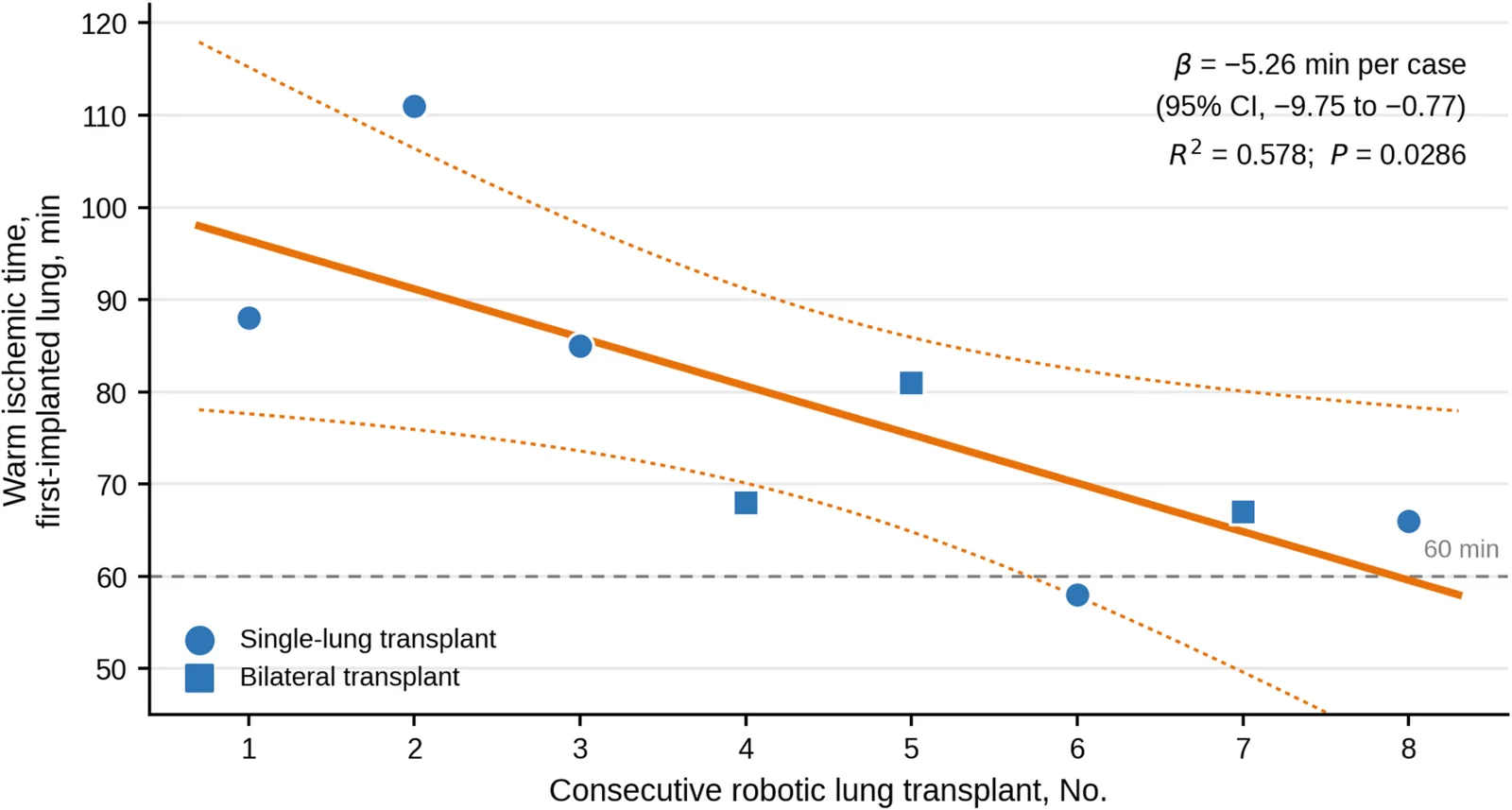
Exploratory post hoc regression of warm ischemic time of the first-implanted lung on consecutive case number across the eight patient-level cases of the first-in-man case series. The solid line is the ordinary least-squares fit and dotted lines the 95% confidence band computed on the small-sample t distribution with 6 residual degrees of freedom: beta = -5.26 min per case (95% CI, -9.75 to -0.77); R2 = 0.578; *P* = 0.0286. Circles denote single-lung and squares bilateral procedures. This unadjusted chronological regression cannot separate accumulating experience from case mix, laterality, workflow change, or platform iteration, and is not a validated learning curve

Across the eight consecutive patient-level cases from the first-in-man case series, warm ischemic time of the first-implanted lung fell significantly with accumulating experience (Fig. 5): the fitted slope was − 5.3 min per consecutive case (95% CI − 9.75 to − 0.77; R² = 0.58, *P* = 0.029), with a mean of 88.0 min across the first four cases versus 68.0 min across the last four. This is consistent with the qualitative observation in the parent cohort that warm ischemic times fell from a maximum of 111 min toward approximately one hour [8, 16]. The analysis is an unadjusted chronological regression in eight cases from one center and cannot separate accumulating experience from case mix, laterality, workflow change, or platform iteration; it is hypothesis-generating and is not a validated learning curve.

### Comparison across all three access strategies

**Fig. 6:**
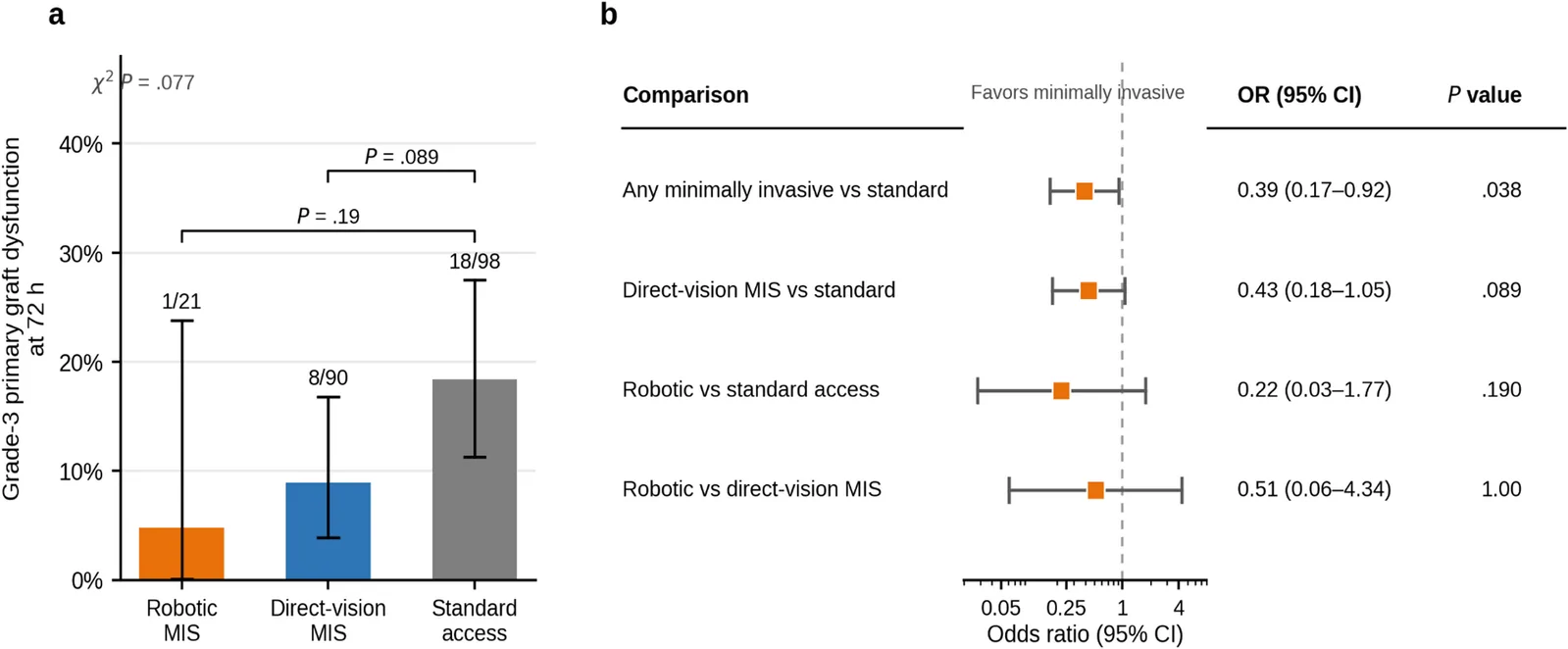
Exploratory post hoc comparison of severe primary graft dysfunction across all three access strategies. **a** Grade-3 primary graft dysfunction at 72 h with exact (Clopper-Pearson) 95% confidence intervals and pairwise two-sided Fisher exact *P* values; the global three-group comparison gave chi-square *P* = 0.077. **b** Unadjusted odds ratios with 95% confidence intervals and Fisher exact *P* values. Access strategy was chosen clinically in a single-center series; the standard-access group was younger (mean 56.9 vs. 66 years) with more intraoperative extracorporeal support. Confounding by indication is substantial, and the pooled comparison is hypothesis-generating and supports no causal claim. *MIS*, minimally invasive surgery; *OR*, odds ratio

The comparative cohort also reported 98 contemporaneous standard-access transplants (sternotomy *n* = 71, clamshell *n* = 19, thoracotomy > 6 cm *n* = 8), permitting a three-way comparison of severe graft dysfunction (Table 8; Fig. 6) [16]. Grade-3 PGD at 72 h occurred in 4.8% of robotic (1/21; exact 95% CI 0.1–23.8), 8.9% of direct-vision minimally invasive (8/90; 3.9–16.8), and 18.4% of standard-access recipients (18/98; 11.3–27.5); the global three-group comparison did not reach significance (χ² *P* = 0.077). Unadjusted odds ratios were 0.22 (95% CI 0.03–1.77; Fisher exact *P* = 0.19) for robotic versus standard access and 0.43 (0.18–1.05; *P* = 0.089) for direct-vision minimally invasive versus standard access. Pooling both minimally invasive strategies against standard access yielded an odds ratio of 0.39 (95% CI 0.17–0.92; Fisher exact *P* = 0.038). This analysis is exploratory and post hoc. This last comparison must be interpreted with great caution: the standard-access group was substantially younger (mean 56.9 vs. 66 years), received intraoperative extracorporeal support more often (43.8% vs. 42.9% and 27.8%), and was selected for open access precisely because of anatomical or physiological complexity—confounding by indication that no unadjusted analysis can remove.

**Table 8:** Severe primary graft dysfunction across all three access strategies (single-center contemporaneous series, *n* = 209)

Access strategy	*n*	Grade-3 PGD at 72 h	Exact 95% CI	Odds ratio vs. standard access (95% CI)
Robotic minimally invasive	21	4.8% (1/21)	0.1–23.8	0.22 (0.03–1.77)
Direct-vision minimally invasive	90	8.9% (8/90)	3.9–16.8	0.43 (0.18–1.05)
Standard access (sternotomy, clamshell, thoracotomy > 6 cm)	98	18.4% (18/98)	11.3–27.5	Reference
Any minimally invasive (pooled)	111	8.1% (9/111)	3.8–14.8	0.39 (0.17–0.92)

Three-group comparison χ² *P* = 0.077; pooled minimally invasive versus standard access Fisher exact *P* = 0.038. These odds ratios are unadjusted and derived from a single-center series in which access strategy was selected clinically: the standard-access group was younger (mean 56.9 vs. 66 years) and had a higher rate of intraoperative extracorporeal support, so confounding by indication is substantial and no causal inference is warranted. One-year survival was 95.0%, 95.5%, and 94.0% respectively. PGD, primary graft dysfunction

### Graft dysfunction trajectory and ischemic time

**Fig. 7:**
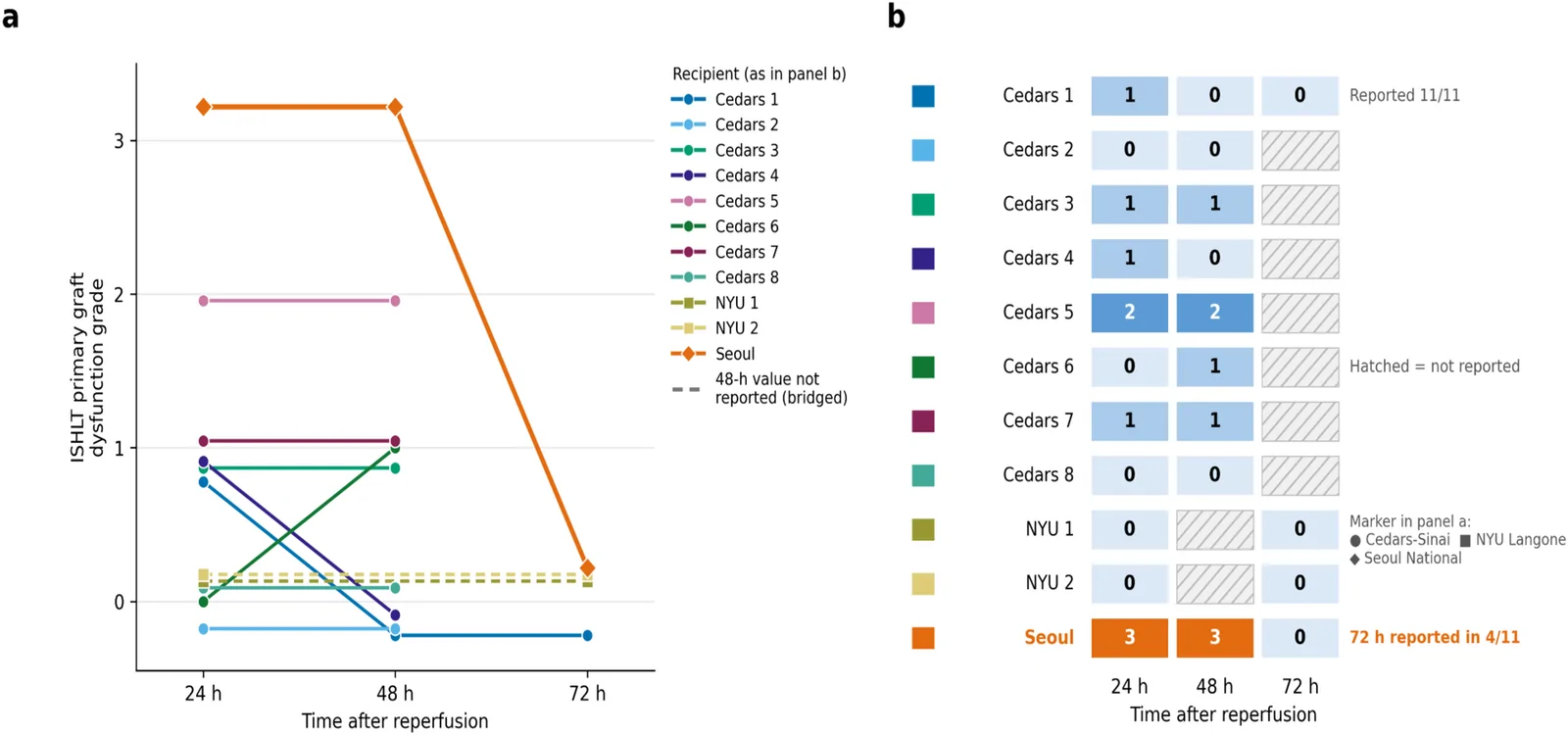
Observed primary graft dysfunction grades and reporting availability. **a** Serial ISHLT grades for the eleven recipients with any serial reporting, plotting observed values only. Each recipient is drawn in a distinct colour and identified in the legend by the same label used in panel b; marker shape denotes the reporting center (circles, Cedars-Sinai; squares, NYU Langone; diamond, Seoul National). Solid segments join consecutive observed values; the dashed segments for the two NYU recipients bridge an unreported 48-hour value between observed 24- and 72-hour grades and do not represent observed data. Points are jittered vertically by a small fixed offset per recipient to separate overlapping trajectories; all plotted values are integer grades. **b** Reporting-availability matrix, with each row colour-keyed to panel a: a 24-hour grade was reported for 11 of 11 recipients, a 48-hour grade for 9 of 11, and a 72-hour grade for only 4 of 11, all grade 0. Hatched cells were not reported; they are not imputed and must not be read as grade 0. Primary graft dysfunction is scored at recipient level, so grades are not displayed per lung. In the eight-patient case series, warm ischemic time did not correlate with 24-hour grade (Spearman rho = 0.26; *P* = 0.53). ISHLT, International Society for Heart and Lung Transplantation

Serial PGD grades were reported for eleven recipients at 24 h, nine at 48 h, and only four at 72 h (Fig. 7). All four recipients with a reported 72-hour value were grade 0, including the assistant-independent Seoul case, which was grade 3 at 24 and 48 h before resolving despite the longest reported warm ischemic time in the literature (131 min) [18]. The seven Cedars-Sinai recipients without a reported 72-hour value are shown as unreported and must not be interpreted as grade 0; the 72-hour finding therefore cannot be generalised beyond those four recipients. Across the eight first-in-man cases, warm ischemic time did not correlate with PGD grade at 24 h (Spearman ρ = 0.26, *P* = 0.53). Cold ischemic times among the four recipients with a reported 72-hour grade spanned 163 to 590 min, and all four were grade 0—including recipients whose lungs were implanted after 491 min of cold storage in the New York series and after 590 min at 10 °C in Seoul [9, 18]. Because primary graft dysfunction is scored at recipient level, ischemic times are not plotted against grade for individual lungs. No statistically significant difference in pulmonary function was detected between the robotic and direct-vision minimally invasive groups at 3, 6, or 12 months in the source repeated-measures mixed-effects model (all *P* > 0.05; Fig. 4). The standard-access series was reported descriptively and was not included in that model, so no formal three-way inference is available [16].

### Technical heterogeneity

**Fig. 8:**
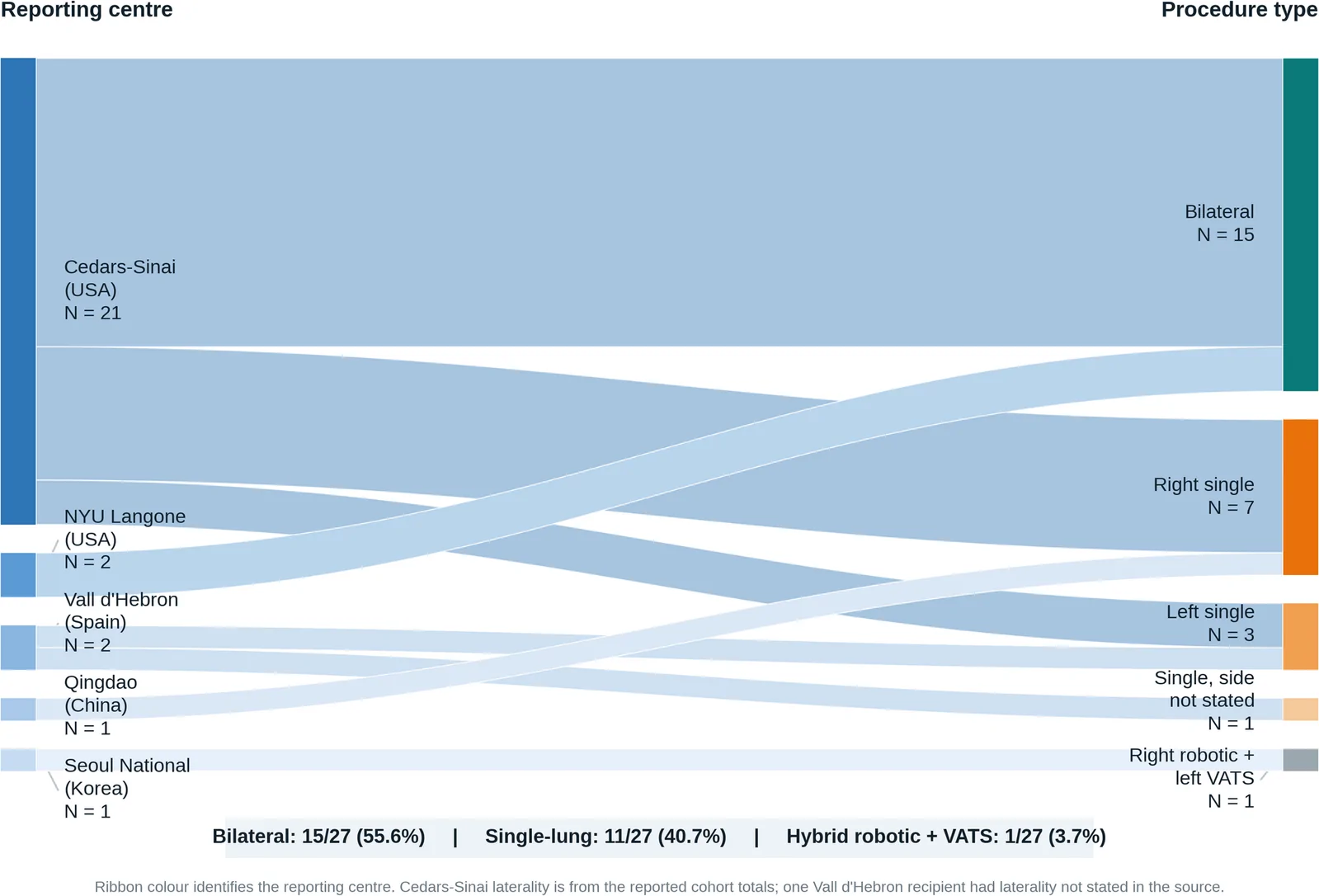
Distribution of procedure type across all 27 reported robotic lung transplant recipients, by reporting center. Ribbon width is proportional to the number of recipients and ribbon colour identifies the reporting center. Cedars-Sinai laterality is taken from the reported cohort totals (13 bilateral, 6 right single, 2 left single) rather than from patient-level data; laterality was not stated for one Vall d’Hebron recipient. The Seoul recipient underwent robotic right implantation with a video-assisted thoracoscopic left implantation and is shown separately. Bilateral procedures accounted for 15 of 27 recipients (55.6%)

**Fig. 9:**
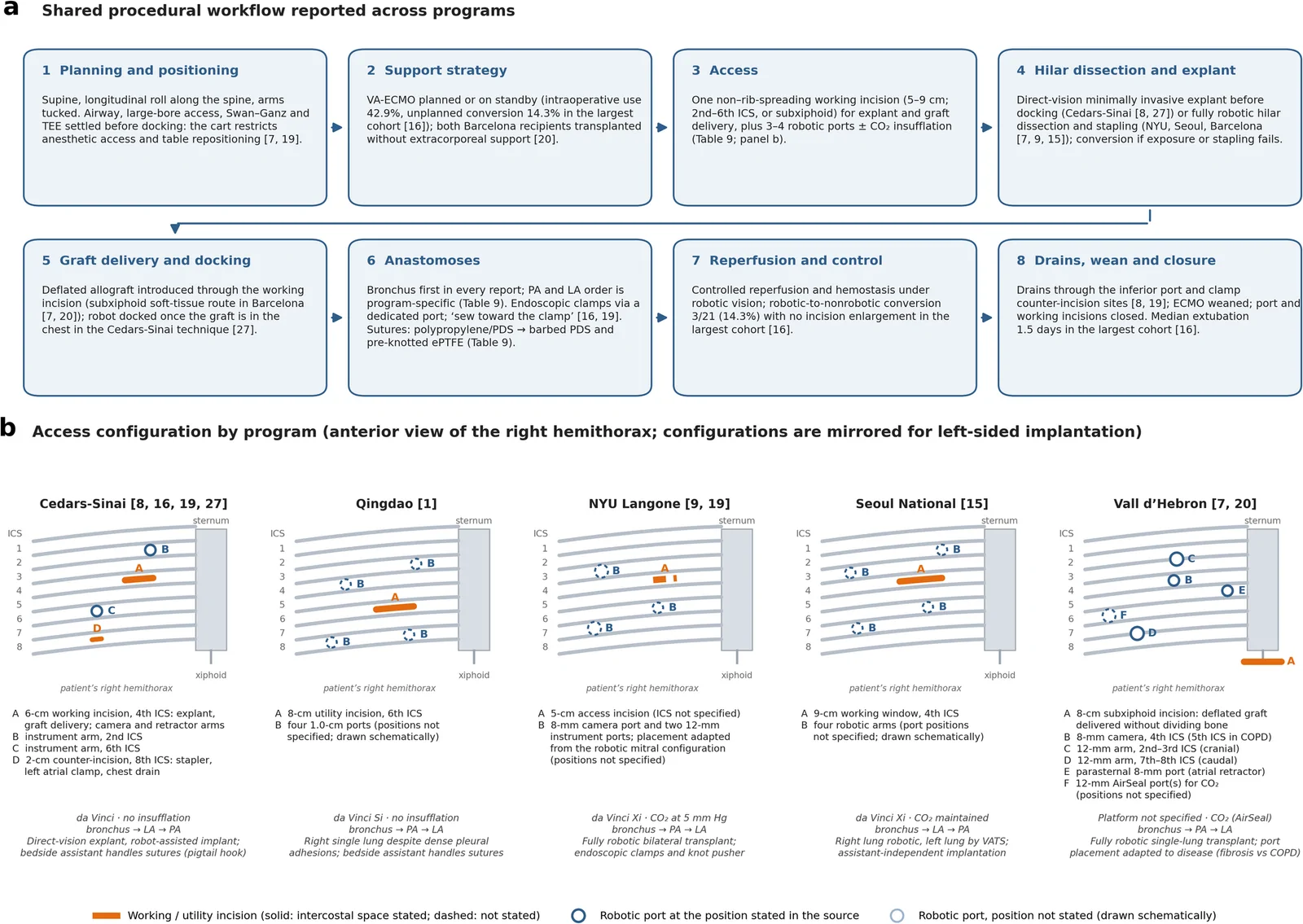
Operative technique of robotic lung transplantation as reported by the five programs. **a** Shared procedural workflow synthesized from the included reports and associated technical descriptions; bracketed numbers are reference citations. **b** Access configuration of each program on an anterior view of the right hemithorax (mirrored for left-sided implantation). Orange bars denote the working or utility incision drawn to approximate scale (solid where the intercostal space was stated in the source, dashed where it was not); circles denote robotic ports (dark outline, position stated in the source; pale dashed outline, position not stated and drawn schematically); letters key each element to the list beneath each panel. The Cedars-Sinai configuration is that of the index reports [8, 20]; the group has since described a third-space working incision with ports in the second, fourth, and fifth spaces [22]. The Vall d’Hebron port scheme is taken from the group’s standardized-technique report [7]. Platform, insufflation, anastomotic sequence, and bedside assistance are tabulated in Table 9. COPD, chronic obstructive pulmonary disease; ECMO, extracorporeal membrane oxygenation; ePTFE, expanded polytetrafluoroethylene; ICS, intercostal space; LA, left atrium; PA, pulmonary artery; PDS, polydioxanone; TEE, transesophageal echocardiography; VATS, video-assisted thoracoscopic surgery

Operative configuration differed substantially across centers (Table 9): access ranged from a 5-cm anterior incision to a 9-cm working window, and one program delivered the graft through an 8-cm subxiphoid incision rather than a thoracotomy; insufflation was used in the two most recent programs and also forms part of the Barcelona group’s standardized technique [7], the anastomotic sequence varied, and bedside assistance ranged from suture handling to a fully assistant-independent technique [1, 8, 9, 16, 18]. No single standardized robotic lung transplant technique yet exists. Procedure type by center is shown in Fig. 8, and the shared operative workflow and the access configuration of each program are summarized schematically in Fig. 9.

**Table 9:** Operative and technical configuration across reporting centers

Center	Platform	Access	Insufflation	Anastomotic sequence	Bedside assistance
Cedars-Sinai [8, 16]	da Vinci	6-cm thoracotomy, 4th ICS (+ 2-cm clamp incision, 8th ICS)	None	Bronchus – left atrium – pulmonary artery	Suture handling with pigtail hook
Qingdao [1]	da Vinci Si	8-cm utility incision, 6th ICS; four 1.0-cm ports	None	Bronchus – pulmonary artery – atrium	Suture handling
NYU Langone [9]	da Vinci Xi	5-cm access incision; 8-mm camera, two 12-mm ports	CO_2_ at 5 mm Hg	Bronchus – pulmonary artery – left atrium	Clamps and knotpusher
Seoul National [15]	da Vinci Xi	9-cm working window, 4th ICS; four arms	CO_2_ maintained	Bronchus – atrium – pulmonary artery	Assistant-independent
Vall d’Hebron [7, 18, 20]	Not specified	8-cm subxiphoid incision; four ports	Not stated for the two recipients [20]; CO_2_ via AirSeal ports in the standardized technique [7]	Bronchus – pulmonary artery – left atrium	Suture handling

Suture selection progressed from conventional polypropylene and polydioxanone (earliest reports) to barbed polydioxanone and preknotted expanded polytetrafluoroethylene (most recent). In the Barcelona standardized technique the 8-mm camera port is placed in the fourth intercostal space for fibrotic and the fifth for emphysematous lungs, 12-mm instrument ports at the second–third and seventh–eighth spaces, and a parasternal 8-mm port carries an atrial retractor; AirSeal ports provide CO_2_ insufflation, with caution advised in hypercapnic recipients [7]. Access configurations are drawn in Fig. 9b. ICS, intercostal space

### Operative workflow

Despite this heterogeneity, the published descriptions share a common procedural sequence, summarized in Fig. 9a [1, 7–9, 16, 18–20]. Recipients are positioned supine with a longitudinal roll along the spine and the arms tucked, and the airway, large-bore vascular access, pulmonary artery catheter, and transesophageal echocardiography are secured before docking, because the robotic cart restricts anesthetic access and prevents table repositioning once docked [7, 19]. The extracorporeal-support strategy is settled in advance: intraoperative veno-arterial ECMO was used in 42.9% of the largest cohort, with unplanned conversion to ECMO in a further 14.3% [16], whereas both Barcelona recipients were transplanted without extracorporeal support [21]. Access comprises a single non–rib-spreading working incision of 5–9 cm, through which the native lung is removed and the deflated allograft delivered, plus three or four robotic ports (Fig. 9b). In the Cedars-Sinai technique the working incision lies in the anterior fourth intercostal space, the native lung is explanted under direct vision before docking, the camera and retractor arms enter through the working incision itself, the instrument arms through the second and sixth intercostal spaces, and a 2-cm counter-incision in the eighth intercostal space accommodates the stapler, the left atrial clamp, and subsequently a chest drain [8, 19, 20]. The Barcelona technique delivers the graft through an 8-cm subxiphoid soft-tissue incision without dividing bone and adapts port placement to the underlying disease [7, 21]. The New York group performed explant and implant fully robotically under low-pressure CO₂ insufflation with endoscopic clamps and a knot pusher [9, 19], with port placement initiated from the group’s robotic mitral configuration according to its meeting abstract (Table 10); the Seoul technique maintained CO₂ insufflation and eliminated bedside suture handling altogether [18]; and the Qingdao case combined an 8-cm utility incision in the sixth intercostal space with four 1.0-cm ports and no insufflation [1]. The bronchial anastomosis was performed first in every report, whereas the order of the arterial and atrial anastomoses is program-specific (Table 9). Vascular control is obtained with endoscopic clamps (Chitwood–DeBakey, robotic or endoscopic Satinsky) introduced through a dedicated port, and the atrial anastomosis is sewn toward the clamp using backhand needle angles [16, 19]. Suture material has moved from conventional polypropylene and polydioxanone to barbed polydioxanone and pre-knotted expanded polytetrafluoroethylene, which removes the need for a bedside assistant to tie knots (Table 9). After reperfusion, drains are placed through the inferior port site and the clamp counter-incision, extracorporeal support is weaned, and the incisions are closed [8, 19]. Port positions continue to evolve within programs—the Cedars-Sinai group has since described a third-space working incision with ports in the second, fourth, and fifth spaces [22]—so Fig. 9b depicts the configurations of the index reports rather than fixed templates.

**Table 10:** Programs identified only through institutional press releases or conference abstracts (narrative context; no outcome data extracted)

Program	Location	Procedure date	Source type	Citation (accessed 16 August 2026)
Cleveland Clinic Abu Dhabi	Abu Dhabi, UAE	By July 2025	Institutional press release	Cleveland Clinic Abu Dhabi performs first robotic lung transplant in the Gulf region. Cleveland Clinic Newsroom, 8 July 2025. Two recipients with idiopathic pulmonary fibrosis and pulmonary hypertension, supported with veno-arterial extracorporeal membrane oxygenation. https://newsroom.clevelandclinic.org/2025/07/08/cleveland-clinic-abu-dhabi-performs-first-robotic-lung-transplant-in-the-gulf-region
Cleveland Clinic	Ohio, USA	May 2026	Institutional press release	Cleveland Clinic completes its first robotic lung transplant in U.S. Cleveland Clinic Newsroom, 24 June 2026. https://newsroom.clevelandclinic.org/2026/06/24/cleveland-clinic-completes-its-first-robotic-lung-transplant-in-us
Baylor St. Luke’s Medical Center	Texas, USA	March 2025	Institutional blog	Loor G, Liao KK, Fernandez R. Robotic lung transplantation and the evolution of surgical options. Baylor College of Medicine Blog Network, 1 May 2025. https://blogs.bcm.edu/2025/05/01/robotic-lung-transplantation-and-the-evolution-of-surgical-options/
Northwestern Medicine, Canning Thoracic Institute	Illinois, USA	August 2025	Institutional press release	Northwestern Medicine Canning Thoracic Institute performs Illinois’ first known robotic lung transplant. Northwestern Medicine Newsroom, 30 September 2025. https://news.nm.org/northwestern-medicine-canning-thoracic-institute-performs-illinois-first-known-robotic-lung-transplant-pioneering-minimally-invasive-approach-for-select-patients/
Three-center collaborative: Cedars-Sinai, NYU Langone, Vall d’Hebron	USA and Spain	2025	Meeting abstract	The Worldwide Robotic Lung Transplantation Experience To-Date: Outcomes in the First Three Centers. Am J Transplant 2025. https://doi.org/10.1016/j.ajt.2025.07.426
Three-center collaborative (companion abstract): Cedars-Sinai, NYU Langone, Vall d’Hebron	USA and Spain	2025	Meeting abstract	The Worldwide Robotic Lung Transplantation Experience To-Date: Approaches and Outcomes. J Heart Lung Transplant 2025. https://doi.org/10.1016/j.healun.2025.02.596
NYU Langone Health (single-center abstract)	New York, USA	2025	Meeting abstract	Early Technical Considerations and Patient Selection in Robotic Lung Transplantation. J Heart Lung Transplant 2025. https://doi.org/10.1016/j.healun.2025.02.588

Records were identified through general web searching and Embase/Web of Science conference indexing. All entries carry a traceable citation with an access date. The three-center collaborative comprises Cedars-Sinai Medical Center (Los Angeles), NYU Langone Health (New York), and Hospital Universitario Vall d’Hebron (Barcelona), identified from the author affiliations of the abstracts; the ATC 2025 abstract reported 34 robotic lung transplants at these centers (23 bilateral, 8 right single, 3 left single). The abstract on technical considerations and patient selection is from NYU Langone Health and reports that all candidates were considered for robotic bilateral transplantation, that operative timing or patient characteristics led to open implantation of the second lung in some, and that one procedure was converted to open for difficulty stapling the hilar structures. Press releases and conference abstracts informed narrative context only and contributed no outcome data to the synthesis, and none was appraised for risk of bias

### Recipient selection

How recipients were chosen for the robotic approach was reported inconsistently (Table 11). Only the Cedars-Sinai program described a selection framework: consecutive robotic and direct-vision minimally invasive recipients entered a prospective registry, the two cohorts had similar risk factors and lung allocation scores, and standard access was reserved for candidates with anatomical or physiological complexity, so the robotic cohort represents clinically selected candidates [16]. The group’s technical guide adds anatomical criteria—a capacious chest cavity, favorable body composition and chest-wall accessibility, and an aorta-to-chest-wall distance compatible with central cannulation [19]. The longer waitlist time of the robotic cohort (50 vs. 22.5 days; *P* = 0.02) is itself consistent with logistic selection [16]. The remaining programs reported indication, laterality, and technique but not the criteria by which recipients were chosen: the Qingdao recipient had dense pleural adhesions that were managed robotically [1], the two New York recipients had emphysema [9], the Seoul recipient had idiopathic pulmonary fibrosis and received the second lung by video-assisted thoracoscopy [18], and the two Barcelona recipients had fibrotic lung disease and were transplanted without extracorporeal support [21]. Prior thoracic surgery, recipient acuity, and pre-transplant bridging support were not described as selection criteria in any included report, and no included recipient was reported to have been bridged to transplantation. Feasibility as described in this literature therefore applies to recipients selected by experienced robotic programs and cannot be assumed to generalize to the broader lung transplant population.

**Table 11:** Recipient selection for the robotic approach and support strategy, by reporting center, as reported

Center	Recipients	Reported basis for selecting the robotic approach	Prior thoracic surgery / adhesions	Bridging support	Intraoperative extracorporeal support
Cedars-Sinai [16, 19]	*n* = 21; pulmonary fibrosis 57.1%; 13 bilateral, 6 right single, 2 left single	Consecutive prospective registry; risk factors and LAS similar to direct-vision minimally invasive recipients; standard access reserved for anatomical or physiological complexity [16]. Technical guide favors a capacious chest cavity, accessible chest wall, and feasible central cannulation [19]	Not reported as a criterion	Not reported	VA-ECMO in 42.9%; unplanned conversion 14.3% [16]
Qingdao [1]	*n* = 1; COPD; right single	Not stated	Dense pleural adhesions present; managed robotically	Not reported	Not reported
NYU Langone [9]	*n* = 2; COPD/emphysema; bilateral	Not stated in the peer-reviewed report; the group’s meeting abstract states that all candidates were considered for robotic bilateral transplantation (Table 11)	Not reported	Not reported	Not reported
Seoul National [15]	*n* = 1; IPF; right robotic + left VATS	Not stated	Not reported	Not reported	Not reported
Vall d’Hebron [7, 20]	*n* = 2; UIP and pulmonary fibrosis; single lung	Not stated for the two recipients; the standardized technique adapts port placement to emphysema versus fibrosis and cautions on CO₂ insufflation in hypercapnia [7]	Not reported	Not reported	None [20]

Not stated/not reported indicates that the item was not addressed in the source publication; absence of a statement should not be read as absence of the feature. Meeting-abstract material (Table 10) informed narrative context only. *COPD*, chronic obstructive pulmonary disease; *IPF*, idiopathic pulmonary fibrosis; *LAS*, lung allocation score; *UIP*, usual interstitial pneumonia; *VA-ECMO*, veno-arterial extracorporeal membrane oxygenation; *VATS*, video-assisted thoracoscopic surgery

## Discussion

This first systematic review of robotic-assisted lung transplantation yields three principal observations. First, across five reporting programs on three continents, the procedure is technically feasible: the bronchial, arterial, and atrial anastomoses can be completed robotically, and the allograft implanted through a small, non–rib-spreading incision [1, 7–9, 18]. Second, in the only cohort with a concurrent comparator, no statistically significant difference in early survival or graft-function outcomes was detected between robotic and established direct-vision minimally invasive transplantation; with 21 robotic recipients, wide confidence intervals, and non-randomized clinical selection, this finding is compatible with equivalence, with modest benefit, and with modest harm alike [16]. Third, this absence of a detectable difference was observed despite significantly longer allograft ischemic times in the robotic arm. Whether the additional ischemic interval imposed by a developing technique is clinically inconsequential cannot be determined from these data: the comparison was neither designed nor powered to detect an ischemia-related increase in early graft injury, and no equivalence margin was prespecified [16].

Indirect evidence suggests that allografts may tolerate longer controlled ischemic intervals than previously assumed, although none of it derives from robotic transplantation. In the Lung Transplant Outcomes Group analysis, grade-3 PGD was driven by donor smoking, cardiopulmonary bypass, single-lung transplantation, recipient obesity, and pulmonary hypertension, not by ischemic time [4]. Controlled hypothermic storage at 10 °C has since extended preservation beyond twelve hours without impairing early outcomes [23]. Whether the longer ischemic times of a nascent robotic workflow are clinically absorbable therefore remains an open question that only prospective evaluation with predefined non-inferiority margins can settle.

Two further observations emerge. First, warm ischemic time fell by about five minutes per consecutive case over the first eight procedures (Fig. 5). Warm ischemia is a candidate auditable metric, but eight unadjusted observations cannot attribute the decline to experience. Second, severe graft dysfunction fell monotonically across the access spectrum, from 18.4% with standard access to 8.9% with direct-vision minimally invasive access to 4.8% with robotic access (Fig. 6). We emphasize that this gradient is unadjusted and confounded by indication—open access is chosen for the most complex recipients—and the robotic-specific comparison remains imprecise (OR 0.22, 95% CI 0.03–1.77). It is nonetheless directionally consistent with the analgesia and recovery benefits already reported for sternal-sparing approaches, and it identifies severe PGD as the outcome that a properly powered prospective comparison should be designed around [2, 16].

The trajectory data are too sparse to refine the ischemia argument. A 72-hour grade was reported for only four of eleven recipients with serial data; all four were grade 0, and one—the recipient with the longest warm ischemic time yet published (131 min)—passed through grade 3 at 24 and 48 h before resolving [18]. No inference can be drawn from a single case about whether early severe PGD is generally recoverable. Ischemic duration alone is nonetheless an inadequate safety endpoint for evaluating this technique [4, 23].

Conversion from a robotic to a nonrobotic approach occurred in about one in seven cases (3/21), with unplanned VA-ECMO in a similar minority, reminders that patient selection and team training are integral to safe adoption [1, 16]. The reported experience derives from recipients selected by experienced programs—explicitly so in the only comparative cohort, where standard access was reserved for complex candidates [16], and by unstated criteria elsewhere (Table 11)—so feasibility in these selected recipients cannot be assumed to extend to candidates with prior thoracic surgery, high acuity, or pre-transplant bridging support, none of which was described among the 27 recipients. Dense adhesions were managed robotically in one case [1] but prompted conversion of the second side to a direct-vision approach in a subsequently reported video case [22], underscoring that anatomical selection criteria remain unresolved. The 2024 Minimally Invasive Organ Transplant Consensus Conference recommendations provide a framework for standardization [2, 11], and preclinical models and stepwise technical descriptions support safety-first adoption [19, 21, 24–26].

**Fig. 10:**
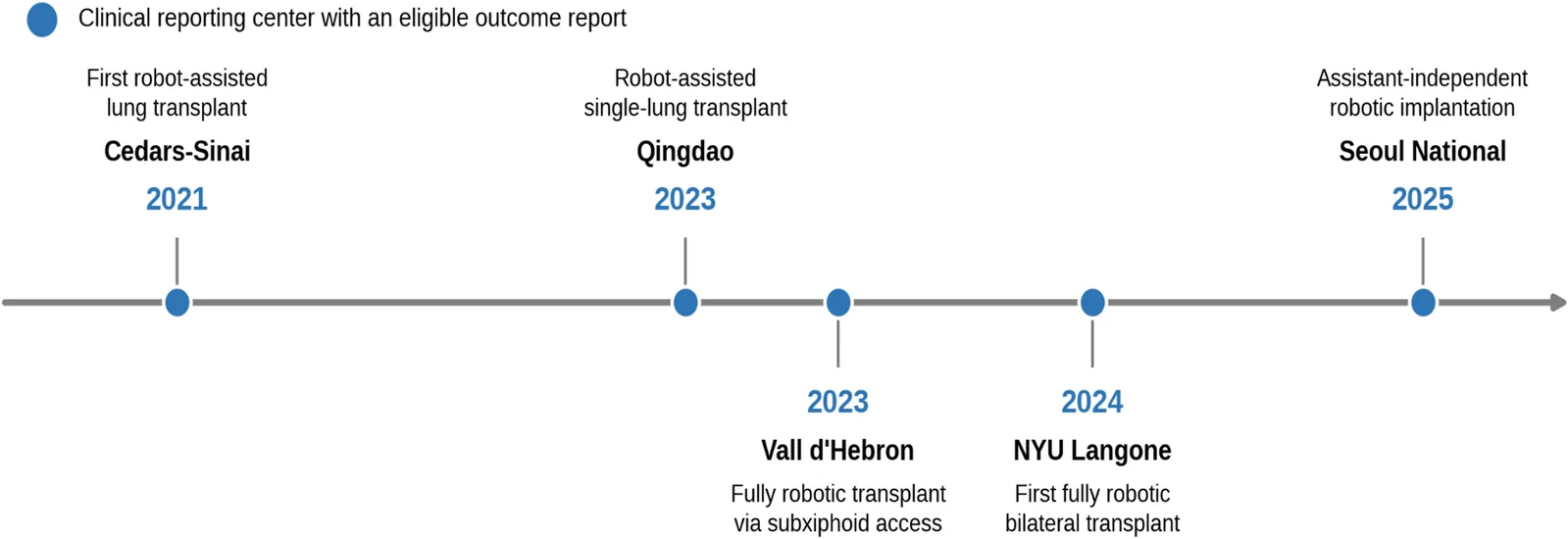
Chronology of reported robotic lung transplantation. Markers denote the five clinical reporting centers with an eligible outcome report: Cedars-Sinai, Qingdao, Vall d’Hebron, New York University, and Seoul National. Barcelona and Qingdao are shown as separate milestones. Dates are of the reported procedure where stated, otherwise of publication

Diffusion is outpacing publication: further centers have performed robotic transplants documented only in press releases or meeting abstracts (Table 10), a three-center collaborative abstract from Cedars-Sinai, NYU Langone, and Vall d’Hebron had accrued 34 procedures by 2025—more than the 27 recipients with peer-reviewed outcome data at our search date—and the chronology spans five years (Fig. 10).

### Limitations

Several limitations warrant emphasis. The literature comprises six reports and 27 recipients, only one with a concurrent comparator; publication and single-center reporting biases are likely. No randomized or prospective comparative data exist, confirmed by the absence of any registered trial in CENTRAL, ClinicalTrials.gov, or the WHO registry. Quantitative pooling was neither valid nor performed, and the computed intervals are wide; absence of a significant difference should not be misread as evidence of equivalence. Design limits causal inference, and certainty was very low for every outcome (Table 4). For the robotic versus direct-vision minimally invasive comparisons shown in Fig. 3, every confidence interval includes unity and no outcome difference was statistically detectable in either direction. This does not extend to the exploratory post hoc pooled comparison against standard access (Table 8). Longer waitlist and ischemic times may reflect selection and logistics intrinsic to early programs. Follow-up is short and chronic outcomes remain unknown. Screening, data extraction, and risk-of-bias appraisal were performed by a single reviewer without independent duplicate assessment; although extracted values were verified against the source publications in a second pass, this departs from PRISMA and Cochrane methodological expectations, and with an evidence base of six reports any single misjudgment of eligibility, extraction, or quality could materially influence the synthesis. Recipient-selection criteria were explicitly reported by only one program (Table 11), so the feasibility summarized here is that of selected recipients at experienced centers. Within these constraints, the principal contribution of this review is not an estimate of effect but a consolidated, critically appraised account of technique, recipient selection, and early outcomes—together with a proposed minimum dataset—for programs weighing adoption.

### Future directions

The central obstacle is not sample size alone but the absence of an agreed outcome set: across the six included reports, none graded airway complications, reported analgesic consumption in comparable units, or reported chronic lung allograft dysfunction. We propose a four-domain minimum dataset for registry reporting (Online Resource 1, S5). First, airway anastomotic complications graded by the ISHLT consensus system [27], the endpoint most sensitive to anastomotic technique, currently unreported. Second, analgesia as cumulative oral morphine milligram equivalents to 72 h and at discharge, with persistent opioid use at 90 days, since reduced pain is the principal claimed advantage of minimal access. Third, chronic lung allograft dysfunction defined and phenotyped by the 2019 ISHLT consensus [28], with CLAD-free survival at three years analysed under competing risks. Fourth, resource and patient-reported measures, including days alive and out of hospital at 90 days. Prospective cohorts with predefined non-inferiority margins remain necessary [23].

## Conclusions

The current evidence, though limited to six early clinical reports, indicates that robotic-assisted lung transplantation is technically feasible across five reporting centers. In the single non-randomized comparative cohort, no difference in perioperative or one-year survival outcomes was statistically detectable, but every robotic versus direct-vision confidence interval includes unity and the analysis is subject to clinical selection. Absence of a detected difference is not evidence of equivalence or of safety. Multicenter, prospective evaluation with standardized outcomes is required before robotic transplantation can be recommended beyond experienced centers.

## Supplementary Information

Below is the link to the electronic supplementary material.

Supplementary Material 1

Supplementary Material 2

## Data Availability

No datasets were generated or analysed during the current study.

## Declarations

### Prior presentation

An abstract based on this work has been accepted for presentation at the 2026 ISMICS Annual Meeting.
