# Supplementary material for: Robotic-assisted lung transplantation: a systematic review of early outcomes in minimally invasive transplant surgery
Source: J Robot Surg. 2026 Sep 19;20(1):1004. doi: 10.1007/s11701-026-03965-7 (PMC13589752; doi:10.1007/s11701-026-03965-7)
Supplement: Supplementary file 1 [file 11701_2026_3965_MOESM1_ESM.docx]

**Supplementary Material**

*Robotic-Assisted Lung Transplantation: A Systematic Review of Early Outcomes in Minimally Invasive Transplant Surgery*

**S1. Electronic search strategies and per-database yields (PRISMA-S)**

*All searches were run from database inception to 15 August 2026 with no language or date restriction. Yields are the number of records retrieved at the time of the search.*

**MEDLINE via PubMed — 112 records**

("Robotic Surgical Procedures"[Mesh] OR "Robotics"[Mesh] OR robotic*[tiab] OR "robot-assisted"[tiab] OR "robot assisted"[tiab] OR robotically[tiab] OR "da Vinci"[tiab] OR telerobotic*[tiab]) AND ("Lung Transplantation"[Mesh] OR "lung transplant*"[tiab] OR "pulmonary transplant*"[tiab] OR "lung allograft*"[tiab] OR "lung graft*"[tiab])

**Embase (Elsevier) — 152 records**

('robot assisted surgery'/exp OR 'robotic surgical procedure'/exp OR 'surgical robot'/exp OR robotic*:ti,ab,kw OR 'robot assisted':ti,ab,kw OR 'da vinci':ti,ab,kw OR telerobotic*:ti,ab,kw) AND ('lung transplantation'/exp OR 'lung graft'/exp OR 'lung transplant*':ti,ab,kw OR 'pulmonary transplant*':ti,ab,kw OR 'lung allograft*':ti,ab,kw)

**Scopus — 80 records**

TITLE-ABS-KEY((robotic* OR "robot-assisted" OR "robot assisted" OR "da Vinci" OR telerobotic*) AND ("lung transplant*" OR "pulmonary transplant*" OR "lung allograft*"))

**Web of Science Core Collection — 48 records**

TS=((robotic* OR "robot-assisted" OR "robot assisted" OR "da Vinci" OR telerobotic*) AND ("lung transplant*" OR "pulmonary transplant*" OR "lung allograft*"))

**Cochrane CENTRAL and CDSR — 1 CENTRAL record**

#1 [mh "Robotic Surgical Procedures"]; #2 (robotic* OR "robot-assisted" OR "da Vinci" OR telerobotic*):ti,ab,kw; #3 #1 OR #2; #4 [mh "Lung Transplantation"]; #5 ("lung transplant*" OR "pulmonary transplant*" OR "lung allograft*"):ti,ab,kw; #6 #4 OR #5; #7 #3 AND #6

**ClinicalTrials.gov and WHO ICTRP — 0 eligible records**

Condition: lung transplantation; Other terms: robotic OR robot-assisted. No registered interventional trial of robotic lung transplantation was identified.

**Other methods — 7 records**

Institutional press releases and blogs identified by general web searching (n = 4) and conference proceedings indexed in Embase and Web of Science (n = 3). Reference lists of included reports and forward-citation searching yielded no additional eligible records. Supplementary academic search engines and Google Scholar were screened without new eligible records.

**S2. De-duplication summary**

*De-duplication was performed on digital object identifiers across the three databases exported in full (Embase, Scopus, Web of Science); records lacking a DOI were matched on normalised title. MEDLINE and CENTRAL records were not exported in a machine-readable format, so overlap between those two sources and the exported databases could not be verified automatically; the duplicate count below therefore reflects the exported databases and is a conservative floor.*

| **Step** | **Records** |
| --- | --- |
| Embase | 152 |
| Scopus | 80 |
| Web of Science | 48 |
| Subtotal, exported databases | 280 |
| — carrying a DOI | 256 |
| — lacking a DOI | 24 |
| Unique DOIs | 169 |
| Duplicates removed by DOI | 87 |
| Unique records lacking a DOI (title-matched) | 17 |
| Duplicates removed by title match | 7 |
| Unique records, exported databases | 186 |
| Total duplicates removed | 94 |
| MEDLINE (PubMed) | 112 |
| Cochrane CENTRAL | 1 |
| All database records identified | 393 |
| Records screened after de-duplication | 299 |
| Records excluded at title/abstract | 291 |
| Full-text reports assessed (databases stream) | 8 |
| Reports identified by other methods | 7 |
| Full-text reports assessed (other methods stream) | 7 |
| Reports excluded | 9 |
| Reports included in synthesis | 6 |

*All five included reports were present in all four core databases.*

**S3. Full-text reports excluded, with reasons (n = 9)**

*Note on a borderline record. Ascanio F, Royo-Crespo I, Jauregui A. Robotic lung transplantation: a paradigm shift in surgical strategy. Arch Bronconeumol 2024;60:3–4 is designated an Editorial by its journal, but reports two human recipients with donor and recipient characteristics, operative technique, and post-operative clinical outcomes. Eligibility was therefore determined on content rather than article-type label, and this report was included in the synthesis. A companion commentary by the same group without patient-level outcomes was excluded.*

| **Report** | **Stream** | **Reason for exclusion** |
| --- | --- | --- |
| Ascanio F, Royo-Crespo I, Rosado J, et al. Advances in robotic lung transplantation: development and validation of a new surgical technique in animal models. Interdiscip Cardiovasc Thorac Surg 2023;37:ivad179 | Databases | Animal (sheep) model; not a human clinical report |
| Ascanio F, Abdallah NZ, Rosado J, et al. Towards standardization in robotic lung transplants. Eur J Cardiothorac Surg 2025;67:ezaf042 | Databases | Standardized-technique report without separately identifiable patient outcomes |
| Cleveland Clinic Abu Dhabi press release (2025) | Other methods | Press release; no eligible clinical outcome data |
| Cleveland Clinic (Ohio) press release (2026) | Other methods | Press release; no eligible clinical outcome data |
| Baylor College of Medicine blog (2025) | Other methods | Institutional blog; no eligible clinical outcome data |
| Northwestern Medicine press release (2025) | Other methods | Press release; no eligible clinical outcome data |
| Worldwide Robotic Lung Transplantation Experience (ATC 2025 abstract) | Other methods | Meeting abstract; not a full-length peer-reviewed report |
| Early Technical Considerations and Patient Selection (ISHLT 2025 abstract) | Other methods | Meeting abstract; not a full-length peer-reviewed report |

**S4. Recomputed comparative estimates**

*Odds ratios computed by the Woolf method with the Haldane–Anscombe correction only where a zero cell was present; P values are two-sided Fisher exact tests recomputed from the displayed counts, without adjustment for multiplicity.*

| **Outcome** | **Robotic** | **Direct MIS** | **OR (95% CI)** | **Fisher P** |
| --- | --- | --- | --- | --- |
| Ventilator support <48 h | 16/21 | 68/90 | 1.04 (0.34–3.15) | 1.00 |
| Grade-3 PGD at 72 h | 1/21 | 8/90 | 0.51 (0.06–4.34) | 1.00 |
| Epidural analgesia | 19/21 | 79/90 | 1.32 (0.27–6.47) | 1.00 |
| Discharged on opioids | 6/19 | 35/90 | 0.73 (0.25–2.09) | .612 |
| Reoperation | 2/20 | 3/90 | 3.22 (0.50–20.69) | .223 |
| Tracheostomy | 1/20 | 3/90 | 1.53 (0.15–15.48) | .557 |
| New dialysis | 0/20 | 2/90 | 0.86 (0.04–18.68) | 1.00 |
| Posttransplant stroke | 0/20 | 2/90 | 0.86 (0.04–18.68) | 1.00 |
| 30-day readmission | 2/20 | 12/90 | 0.72 (0.15–3.51) | 1.00 |
| 30-day death | 1/20 | 0/90 | 13.92 (0.55–354.75) | .182 |

*All ten confidence intervals include unity. For 30-day death the source publication reports P = 0.03; the exact test on the displayed counts gives P = 0.182.*

**S5. Proposed minimum dataset for multicenter reporting of robotic lung transplantation**

*Derived from the reporting gaps identified in this review. Across the six included reports, no study graded airway complications, none reported analgesic consumption in comparable units, and none reported chronic lung allograft dysfunction. Consensus instruments are cited where they exist; where none exists, the proposed unit is given.*

**Domain 1. Technique and intraoperative course**

| **Variable** | **Unit or definition** |
| --- | --- |
| Access | Incision type, length (cm), and site (intercostal space or subxiphoid) |
| Ports | Number, size, and intercostal position |
| Insufflation | Used or not; pressure (mm Hg) |
| Anastomotic sequence | Order of bronchial, arterial, and atrial anastomoses |
| Suture material | Type and calibre for each anastomosis |
| Bedside assistance | Conventional suture handling, knot-pusher, or assistant-independent |
| Console and total operative time | Minutes |
| Cold and warm ischemic time | Minutes, reported per implanted lung |
| Extracorporeal support | None, planned VA-ECMO, unplanned VA-ECMO, or cardiopulmonary bypass |
| Conversion | Robotic to nonrobotic; robotic to open anastomosis; incision enlargement; with reason |

**Domain 2. Graft function and airway integrity**

| **Variable** | **Instrument or definition** |
| --- | --- |
| Primary graft dysfunction | ISHLT grade at 0, 24, 48, and 72 h; time to resolution |
| Airway anastomotic complications | ISHLT 2018 consensus grading: ischemia, necrosis, dehiscence, stenosis, malacia |
| Airway intervention | Balloon dilation, stent, or reoperation; time to first intervention |
| Acute cellular rejection | ISHLT A- and B-grades on surveillance or for-cause biopsy |
| Antibody-mediated rejection | ISHLT criteria; de novo donor-specific antibody class and mean fluorescence intensity |
| Pulmonary function | FEV1 and FVC (% predicted) at 3, 6, and 12 months, then annually; also as % of best post-transplant baseline |
| Chronic lung allograft dysfunction | 2019 ISHLT consensus definition and phenotype (BOS, RAS, mixed, undefined); date of onset |

**Domain 3. Recovery, analgesia, and morbidity**

| **Variable** | **Unit or definition** |
| --- | --- |
| Analgesic consumption | Cumulative oral morphine milligram equivalents at 24, 48, and 72 h and at discharge |
| Regional technique | Epidural, paravertebral, or erector spinae plane block |
| Pain scores | Numerical rating scale at rest and with cough, daily to day 3 |
| Persistent opioid use | Any opioid prescription at 90 days |
| Chest wall and phrenic | Wound infection, dehiscence, osteomyelitis; phrenic nerve injury or diaphragmatic dysfunction |
| Ventilation | Duration (h); proportion supported for less than 48 h; tracheostomy |
| Renal | Acute kidney injury by KDIGO stage; renal replacement therapy |
| Infection | Cytomegalovirus viraemia or disease; invasive fungal infection; ventilator-associated pneumonia; empyema |
| Other | Atrial fibrillation, stroke, delirium, reoperation for bleeding, transfusion volume |

**Domain 4. Survival, resource use, and patient-reported outcomes**

| **Variable** | **Unit or definition** |
| --- | --- |
| Survival | Patient survival and graft survival censored at retransplantation, at 30 d, 1 y, and 3 y |
| CLAD-free survival | Three-year composite, analysed under competing risks (death as competing event) |
| Composite recovery | Days alive and out of hospital at 90 days |
| Length of stay | Intensive care and total hospital days; discharge destination |
| Readmission | 30-day and 90-day, all-cause and transplant-related |
| Patient-reported outcomes | St George’s Respiratory Questionnaire; SF-36 or EQ-5D-5L; return to work |
| Function | Six-minute walk distance at 3, 6, and 12 months |

*Analytic notes. Because death precludes chronic lung allograft dysfunction, cumulative incidence should be estimated with the Fine–Gray subdistribution hazard rather than Kaplan–Meier. Comparative analyses of small early cohorts should prespecify non-inferiority margins rather than test for superiority, and should report effect estimates with confidence intervals rather than P values alone.*
