# Supplementary material for: Robotic-assisted lung transplantation: a systematic review of early outcomes in minimally invasive transplant surgery
Source: J Robot Surg. 2026 Sep 19;20(1):1004. doi: 10.1007/s11701-026-03965-7 (PMC13589752; doi:10.1007/s11701-026-03965-7)
Supplement: Supplementary file 2 [file 11701_2026_3965_MOESM2_ESM.docx]

**PRISMA 2020 Checklist**

*Robotic-Assisted Lung Transplantation: A Systematic Review of Early Outcomes in Minimally Invasive Transplant Surgery*

*Location reported refers to the section of the submitted manuscript, its tables and figures, or Online Resource 1.*

| **Item** | **Checklist item** | **Location reported** |
| --- | --- | --- |
| TITLE |  |  |
| 1 | Title identifies the report as a systematic review | Title |
| ABSTRACT |  |  |
| 2 | Structured abstract (PRISMA 2020 for Abstracts) | Structured Abstract |
| INTRODUCTION |  |  |
| 3 | Rationale for the review in the context of existing knowledge | Introduction, paragraphs 1–3 |
| 4 | Explicit statement of the objective(s) or question(s) | Introduction, final paragraph; Abstract (Objective) |
| METHODS |  |  |
| 5 | Eligibility criteria and how studies were grouped | Methods, Eligibility criteria |
| 6 | Information sources, with date last searched | Methods, Information sources and search strategy; Online Resource 1 S1 |
| 7 | Full search strategy for all databases and registers | Online Resource 1 S1; Appendix S1 |
| 8 | Selection process, including number of reviewers and independence | Methods, Study selection and data extraction (single reviewer; duplicate screening not undertaken) |
| 9 | Data collection process, including number of reviewers | Methods, Study selection and data extraction |
| 10a | Outcomes sought and how results were located | Methods, Study selection and data extraction; Tables 2, 3, 7 |
| 10b | Other variables sought | Methods; Table 7; Table 9 |
| 11 | Risk-of-bias assessment: tools, number of reviewers | Methods, Risk of bias assessment; Tables 4A, 4B |
| 12 | Effect measures used | Methods, Synthesis and statistical methods (odds ratios, Woolf 95% CI) |
| 13a | Processes for deciding eligibility for each synthesis | Methods, Synthesis and statistical methods |
| 13b | Data preparation and conversions | Methods, Synthesis and statistical methods; Table 2 footnote |
| 13c | Tabulation and visual display methods | Tables 1–9; Figures 1–8 |
| 13d | Synthesis methods; rationale for not meta-analysing | Methods, Synthesis and statistical methods (only one comparative cohort; no pooling) |
| 13e | Heterogeneity exploration | Not applicable — no pooled estimate |
| 13f | Sensitivity analyses | Not performed; stated in Methods |
| 14 | Reporting bias assessment | Discussion, Limitations (publication and single-centre reporting bias) |
| 15 | Certainty assessment | Methods; Table 5 (GRADE, by outcome) |
| RESULTS |  |  |
| 16a | Records identified, screened, and included, with flow diagram | Results, Study selection; Figure 1 |
| 16b | Records excluded at full text, with reasons | Figure 1; Online Resource 1 S3 |
| 17 | Citation of each included study | Table 1; References 1, 8, 9, 15, 16 |
| 18 | Risk of bias in each included study | Tables 4A, 4B; Methods (ROBINS-I for the comparative cohort) |
| 19 | Results of each individual study | Tables 1, 2, 3, 7; Figures 2, 3, 6 |
| 20a | Characteristics and risk of bias among contributing studies | Tables 1, 4A, 4B |
| 20b | Results of syntheses | Results; Tables 2, 3, 8; Figures 2–7 |
| 20c | Investigations of causes of heterogeneity | Not applicable — no pooled estimate |
| 20d | Sensitivity analyses | Not performed |
| 21 | Reporting bias assessment results | Discussion, Limitations |
| 22 | Certainty of evidence results | Table 5; Discussion |
| DISCUSSION |  |  |
| 23a | Interpretation in the context of other evidence | Discussion, paragraphs 1–4 |
| 23b | Limitations of the evidence | Discussion, Limitations |
| 23c | Limitations of the review processes | Discussion, Limitations (single-reviewer screening) |
| 23d | Implications for practice, policy, and research | Discussion, Future directions; Conclusions |
| OTHER INFORMATION |  |  |
| 24a | Registration and protocol | Methods, Protocol and reporting; title page (PROSPERO CRD420261479535) |
| 24b | Where the protocol can be accessed | Title page; PROSPERO record |
| 24c | Amendments to registered information | Post hoc analyses identified as not part of the registered protocol (Methods) |
| 25 | Support and sponsorship | Title page, Declarations (Funding) |
| 26 | Competing interests | Title page, Declarations |
| 27 | Availability of data, code, and other materials | Title page, Declarations; Online Resource 1 |

*Prepared in accordance with Page MJ, McKenzie JE, Bossuyt PM, et al. The PRISMA 2020 statement: an updated guideline for reporting systematic reviews. BMJ 2021;372:n71.*
